# Molecular Basis for Specific Regulation of Neuronal Kinesin-3 Motors by Doublecortin Family Proteins

**DOI:** 10.1016/j.molcel.2012.06.025

**Published:** 2012-09-14

**Authors:** Judy S. Liu, Christian R. Schubert, Xiaoqin Fu, Franck J. Fourniol, Jyoti K. Jaiswal, Anne Houdusse, Collin M. Stultz, Carolyn A. Moores, Christopher A. Walsh

**Affiliations:** 1 Center for Neuroscience Research, Children's National Medical Center, Washington, DC 20010, USA; 2 Division of Genetics, Howard Hughes Medical Institute, Manton Center for Orphan Diseases, Children's Hospital Boston, and Department of Pediatrics and Department of Neurology, Harvard Medical School, Boston, MA 02115, USA; 3 Institute of Structural and Molecular Biology, Birkbeck College, London WC1E 7HX, UK; 4 Cancer Research UK London Research Institute, Lincoln's Inn Fields Laboratories, 44 Lincoln's Inn Fields, London, WC2A 3LY, UK; 5 Center for Genetic Medicine Research, Children's National Medical Center, Washington, DC 20010, USA; 6 Structural Motility, Institut Curie, Centre National de la Recherche Scientifique, Unité Mixte de Recherche 144, 75248 Paris Cedex 05, France; 7 Research Laboratory of Electronics and Department of Electrical Engineering and Computer Science, Harvard-MIT Division of Health Sciences and Technology, Massachusetts Institute of Technology, Cambridge, MA 02139, USA

## Abstract

Doublecortin (Dcx) defines a growing family of microtubule (MT)-associated proteins (MAPs) involved in neuronal migration and process outgrowth. We show that Dcx is essential for the function of Kif1a, a kinesin-3 motor protein that traffics synaptic vesicles. Neurons lacking Dcx and/or its structurally conserved paralogue, doublecortin-like kinase 1 (Dclk1), show impaired Kif1a-mediated transport of Vamp2, a cargo of Kif1a, with decreased run length. Human disease-associated mutations in Dcx's linker sequence (e.g., W146C, K174E) alter Kif1a/Vamp2 transport by disrupting Dcx/Kif1a interactions without affecting Dcx MT binding. Dcx specifically enhances binding of the ADP-bound Kif1a motor domain to MTs. Cryo-electron microscopy and subnanometer-resolution image reconstruction reveal the kinesin-dependent conformational variability of MT-bound Dcx and suggest a model for MAP-motor crosstalk on MTs. Alteration of kinesin run length by MAPs represents a previously undiscovered mode of control of kinesin transport and provides a mechanism for regulation of MT-based transport by local signals.

## Highlights

► Dcx is required for neuronal transport mediated by the kinesin-3 motor Kif1a ► Dcx increases Kif1a/Vamp2 run length without affecting conventional kinesin ► Dcx enhances the affinity of the ADP-bound Kif1a motor domain for microtubules ► Kif1a microtubule binding requires displacement of the flexible Dcx domain linker

## Introduction

Microtubule (MT)-based transport uses molecular motors to carry cargos over long cellular distances within neurons. The large number of MT motors, especially kinesins (encoded by 45 genes in humans) with diverse cargo specificities, provides a potential means of fine regulation of trafficking (Caviston and Holzbaur, 2006), but it is not fully understood how MT-based transport systems achieve specificity with regard to cargo load and targeted transport to specific domains within the neuron. Interaction with MT-associated proteins (MAPs) has been proposed as one means to target transport through complex neuronal structures (Jacobson et al., 2006; Shahpasand et al., 2008), as some MAPs show spatially restricted localization in either dendrites or axons (Binder et al., 1986; Dehmelt and Halpain, 2005). The molecular basis, potential regulatory impact, and degree of specificity of such MAP-motor crosstalk at the MT surface, however, are unknown.

Mutations in a gene encoding an unusual MAP, doublecortin (*Dcx*), cause a neuronal migration disorder leading to intellectual disability and epilepsy (des Portes et al., 1998; Gleeson et al., 1998). *Dcx* and related doublecortin domain protein genes, including *doublecortin-like kinase 1* (*Dclk1*), encode proteins with tandem MT binding domains, referred to as N-DC (or R1) and C-DC (or R2) (Coquelle et al., 2006; Kim et al., 2003; Sapir et al., 2000; Taylor et al., 2000), but the exact role of each of these dual domains is unknown. In contrast to other MAPs that bind directly on the surface of the MT protofilament, existing evidence demonstrates Dcx binding in the recess between protofilaments (Fourniol et al., 2010; Moores et al., 2004).

Although the migratory disruption caused by mutations in *Dcx* has widely been regarded as a defect in cytoskeletal regulation (Bielas et al., 2007; Gleeson et al., 1999), Dcx/Dclk1-deficient neurons also show defects in the transport of presynaptic vesicles (Deuel et al., 2006) in the absence of comparable defects in MT organization. The transport deficiency suggests an attractive alternative hypothesis, that Dcx/Dclk1 may regulate transport of membrane and cellular components, perhaps through kinesin motor proteins, and that specific trafficking of membrane constituents to various neuronal domains may in turn regulate cell shape, as well as the presentation of guidance molecules.

Here we show that Dcx/Dclk1-deficient neurons have unexpectedly specific defects in Kif1a-mediated transport of presynaptic vesicles, and that RNAi knockdown of Kif1a in neurons mimics several effects of Dcx/Dclk1 deficiency. We demonstrate specific increases of Kif1a MT binding and run length mediated by Dcx, and our subnanometer structural analysis of the Dcx:MT:kinesin complex suggests a model for how Dcx and Dclk1 facilitate Kif1a-MT association to regulate MT-based transport of cellular components. Our findings thus suggest a mechanism in which local control of Dcx-MT binding might in turn regulate kinesin-based transport of cellular components in developing and adult neurons.

## Results

### Kif1a Is Mislocalized in Dcx/Dclk1-Deficient Neurons

**Figure 1:**
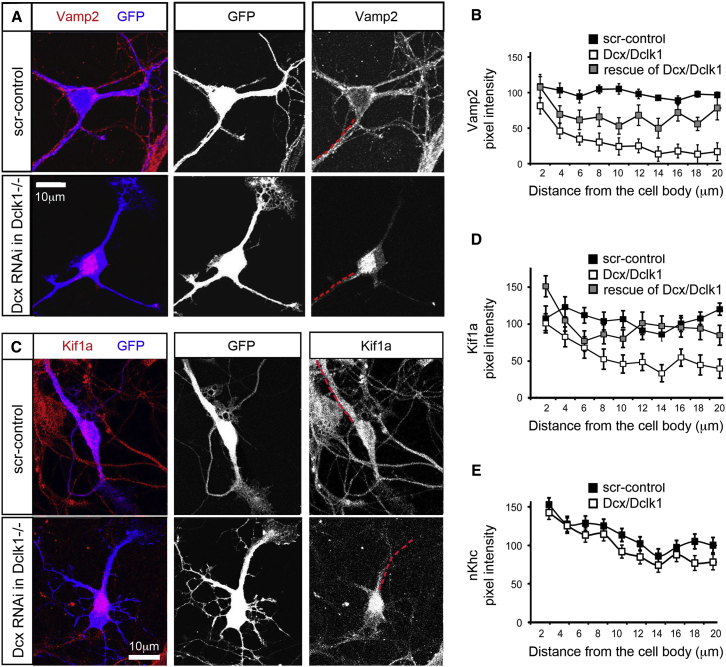
Kif1a Is Mislocalized in Dcx/Dclk1-Deficient Neurons (A) Dissociated WT and *Dclk1*^−/−^ hippocampal neurons are transfected with either a scrambled control shRNAi or a Dcx shRNAi plasmid with a GFP reporter and immunostained for Vamp2 after 4 DIV. (B) Quantification of Vamp2 intensity along the trajectories of neural processes starting from the soma and extending out 20 μm (shown as a broken red line adjacent to the neurite in A) demonstrates statistically significantly (p < 0.05) lower levels of Vamp2 starting at 4 μm from the cell-body Dcx/Dclk- deficient neurons (n = 32) as compared with control (n = 29) in one representative experiment out of four. The Vamp2 level in neurites is partially restored by expression of the shRNAi-resistant HA-Dcx (p < 0.05, n = 23). (C) WT or *Dclk1*^−/−^ neurons are transfected with a plasmid for GFP expression to mark neuronal morphology and shRNAi specific for Dcx where indicated. (D) The pixel intensity of Kif1a versus distance from the cell body of the neuron is shown for WT and Dcx RNAi-treated *Dclk1*^−/−^ neurons, demonstrating significantly less Kif1a neurites of Dcx/Dclk1-deficient neurons after 4 μm from the cell body, and is partially restored by rescue by overexpression of Dcx (n = 37, 31, and 25, respectively). (E) The pixel intensity of neuronal kinesin heavy chain (nKhc) versus distance from the cell body of the neuron is shown for WT and Dcx shRNAi-treated *Dclk1*^−/−^ neurons demonstrating no change in Dcx/Dclk1-deficient neurons (n = 30 and 32, respectively). Error bars represent the standard error of the mean (SEM). Scale bars in all panels represent 10 μm.

Although overexpression of Dcx and Dclk1 induces MT polymerization and sometimes bundling (Bielas et al., 2007; Gleeson et al., 1999; Horesh et al., 1999; Lin et al., 2000), we found that absence of Dcx and Dclk1 does not impact MT organization (data not shown) but instead resulted in defective Vamp2 localization in neurons. Pursuing a previous observation that the presynaptic vesicle protein Vamp2 failed to localize normally at 7 days in vitro (DIV) in Dcx/Dclk1-deficient axons (Deuel et al., 2006), we tested whether Vamp2 was mislocalized in dendrites as well (Song et al., 2009; Tsai et al., 2010). Indeed, Vamp2 was retained in the cell body with defects in axonal and dendritic transport (Figure 1A), which are rescued through expression of an shRNAi-resistant, HA-tagged human Dcx construct (Figure 1B).

Since Vamp2 localization requires trafficking by molecular motors, we examined the distribution of candidate kinesin motors for Vamp2 transport in Dcx/Dclk1-deficient neurons, including conventional kinesin (Song et al., 2009) and Kif1a, a kinesin-3 family motor that transports presynaptic vesicles (Okada and Hirokawa, 1999; Yonekawa et al., 1998). We found that in Dcx/Dclk1-deficient neurons, Kif1a, but not conventional kinesin, is strikingly mislocalized. In wild-type (WT) neurons, Kif1a is present in the cell body and throughout the neurites, whereas Dcx/Dclk1-deficient neurons have less Kif1a in neurites, while the cell body is brightly immunoreactive (Figure 1C). Quantitative immunofluorescence confirms loss of Kif1a staining from Dcx/Dclk1-deficient neurites >4 μm from the cell body, compared to control cells, and this loss can be rescued through expression of an shRNAi-resistant, HA-tagged human Dcx (Figures 1C and D, see Figure S1A available online). In contrast to Kif1a, immunostaining for conventional kinesin reveals no difference between WT and neurons deficient for Dcx/Dclk1 (Figure 1E, Figure S1B), suggesting that Dcx/Dclk1 specifically regulates Kif1a localization.

### Vamp2 Vesicles Are Cargo for the Kinesin-3 Motor Kif1a

**Figure 2:**
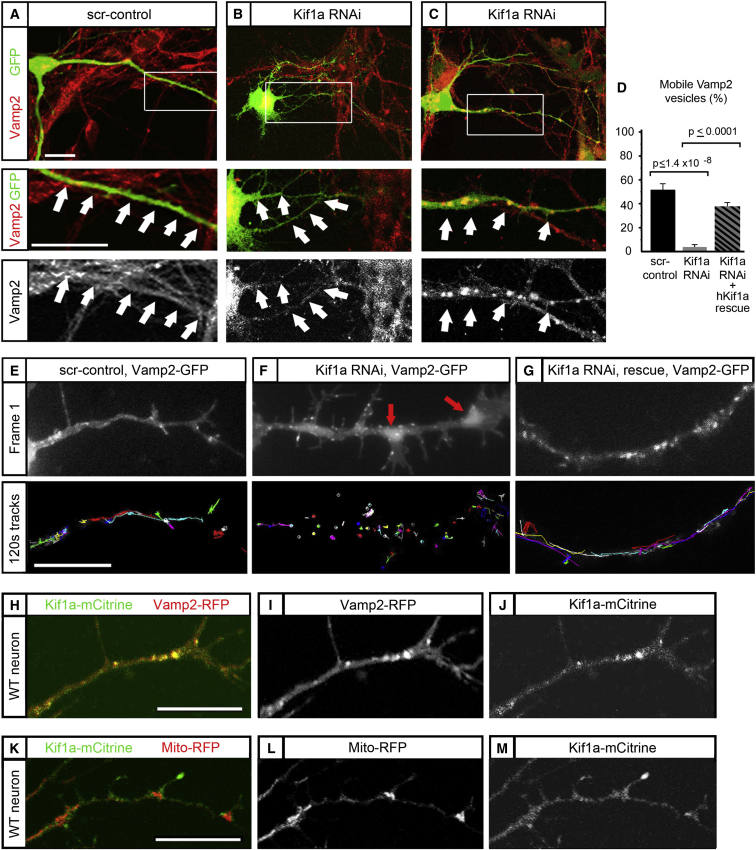
Vamp2 Transport from the Cell Body into Neurites Is Dependent on Kif1a (A)–(C) show DIV4 neurons with high power views (middle/bottom panels) of neurites with both Vamp2 immunostaining (red) and GFP (green), a marker of successful transfection with the shRNAi construct. (A) WT neurons are transfected with a scrambled control showing Vamp2 in neurite of the green cell (white arrows). (B) Knockdown of Kif1a by RNAi results in a majority of neurons with Vamp2 only in the cell body. High-power views (middle/bottom panels) show lack of Vamp2 in the neurites (white arrows). (C) Kif1a knockdown neurons where Vamp2 is clumped in the neurites (white arrows). (D) Quantification of Vamp2-GFP vesicles that moved more than three microns over 120 s are shown for control (56%), knockdown neurons (4%), and rescue (37%). Error bars represent SEM. (E–G) Top panels are the first frame of a 120 s time-lapse video of Vamp2-GFP in control, Kif1a knockdown neurons, and rescue neurons. Bottom panels are the tracking of the Vamp2-GFP. Each color represents the track of a single Vamp2 vesicle over the full 120 s. (H–M) (H) Colocalization of Vamp2-RFP and Kif1a-mCitrine is shown in a DIV5 WT neuron. (I) depicts the Vamp2-RFP channel and (J) the Kif1a-mCitrine channel. (K) Minimal colocalization of Mito-RFP and Kif1a-mCitrine is shown in a DIV5 WT neuron with (L) depicting Mito-RFP and (M) Kif1a-mCitrine. Scale bars, 10 μm.

The mislocalization of Kif1a seen in Dcx/Dclk1-deficient neurons may account for defective Vamp2 localization, since knockdown of Kif1a itself causes very similar defects in Vamp2 localization. Using shRNAi sequences targeting Kif1a (Tsai et al., 2010) (Figure 2, Figure S2), we found that in most Kif1a knockdown neurons, Vamp2 expression was confined to the cell body (Figure 2B, in contrast to normal neurons shown in Figure 2A). A subset of Kif1a knockdown neurons demonstrated abnormally large accumulations of Vamp2 vesicles in neurites (Figure 2C), a defect also classically observed in transport failure (Duncan and Goldstein, 2006), thus strongly suggesting defective transport of Vamp2 in the absence of Kif1a. Live-cell imaging in Kif1a knockdown cells showed near-total loss of observable mobility for Vamp2-GFP (Figures 2D–2G, Movie S1) in both anterograde and retrograde directions, with the number of mobile vesicles being <5% for the knockdown compared with control (Figure 2D), which is consistent with previous reports that Kif1a knockdown decreases bidirectional cargo transport in neurons (Lo et al., 2011). This phenotype could be rescued through expression of an shRNAi-resistant, Myc-tagged human Kif1a (Xue et al., 2010) (Figure 2D), strongly suggesting that Kif1a is involved in the transport of Vamp2. We confirmed the close physical relationship between Kif1a and Vamp2 through coexpression of Kif1a-mCitrine (Hammond et al., 2009) with Vamp2-RFP, showing that the Kif1a motor colocalizes extensively with Vamp2 (Figures 2H–2J). While all of the vesicular Vamp2-RFP appears to colocalize with Kif1a, a fraction of Kif1a-mCitrine is associated with non-Vamp2-positive vesicular structures, suggesting that the motor is also associated with other cargos (Lo et al., 2011). In contrast, coexpression of Kif1a-mCitrine with the mitochondrial marker Mito-RFP shows little colocalization of the motor with mitochondria (Figures 2K–2M), which in neurons are transported by Kif1b (Nangaku et al., 1994; Wozniak et al., 2005) and conventional kinesin (Cho et al., 2007; Glater et al., 2006). Our results therefore suggest that, at the developmental stages examined, Kif1a is the major motor for Vamp2 trafficking and that other motors, such as conventional kinesin, play only a minor role.

### Dcx/Dclk1 and Kif1a Are Essential for Neuronal Migration and Process Outgrowth

Since our findings that Dcx/Dclk1 deficiency disrupts Kif1a localization imply that Kif1a may mediate Dcx function, we investigated whether neurons deficient for Kif1a show defects in migration and morphology similar to those seen in Dcx/Dclk1-deficient neurons. shRNAi constructs targeting Kif1a (Figure S2A) or Dcx were electroporated into the cortex of E15.5 WT and *Dclk1*^*−/−*^ embryos, respectively, after microinjection into the lateral ventricles. While control slice cultures maintained for 4 DIV showed GFP-labeled neurons demonstrating significant migration into the cortical plate (CP), we found that shRNAi constructs disrupting Kif1a protein expression (Figures S2A and S2B) phenocopy Dcx/Dclk1 deficiency in their effects on neuronal migration (Figure S2C), consistent with previous results (Tsai et al., 2010). At the cellular level, Dcx/Dclk1 deficiency and Kif1a knockdown have similar effects on neuronal process length and polarity, with an overall decrease in neurite length when measuring all processes (Figure S2G), a reduction in length in the three longest neurites (Figure S2H), and finally an increase in the number of primary neurites directly arising from the soma (Figure S2I). These changes likely reflect an early disruption of polarization of the neuroblast and are potentially causative for the migration defects observed, and thus Kif1a deficiency appears to closely phenocopy Dcx/Dclk1 deficiency in early stages of cortical development.

### Dcx/Dclk1-Deficient Neurons Have Selective Defects in Vamp2 Vesicle Transport from the Cell Body into Neurites

**Figure 3:**
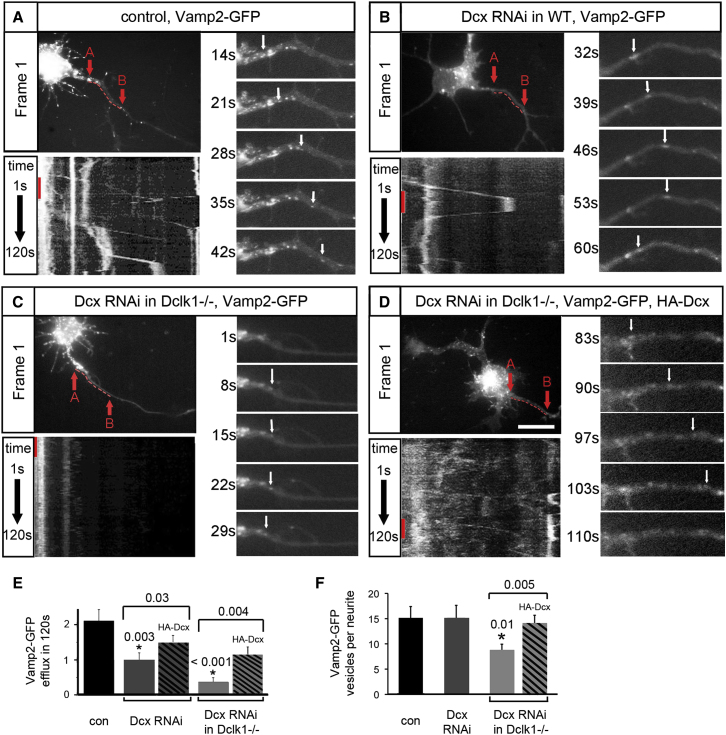
Efflux of Vamp2-GFP into Neurites Is Impaired in Dcx/Dclk1-Deficient Neurons (A–D) Shown is live-cell imaging of control, Dcx-deficient, Dcx/Dclk1-deficient, and HA-Dcx rescue of Dcx/Dclk1 deficient neurons. In (A)–(D), the top left panel is the first frame of the imaging study. A red, broken line 10 μm in length shows the region of the neurite used for generating the kymograph in the bottom left panel. The kymograph is created using the pixels selected by the tracing of the neurite from point A to point B. A red line on the left marks the 28 s time interval depicted by frames in the panel on the right. The right panels show frames at 7 s intervals of the 10 μm region of interest. White arrows mark the position of Vamp2-GFP transport packets. Scale bars, 10 μm in all panels. (E) Quantification of efflux (vesicle exit of the cell body into the neurite) is shown for control, Dcx RNAi, Dcx RNAi in *Dclk1*^−/−^ neurons and Dcx RNAi in *Dclk1^−/−^* with rescue by expression of HA-Dcx. (F) A determination of the number of Vamp2-GFP transport packets seen in (A)–(D) shows a significant decrease in the number of Vamp2-GFP vesicles in the Dcx/Dclk1 double deficient neurons, which can be rescued by expression of HA-Dcx. Error bars in (E) and (F) respresent SEM.

Since Kif1a and Vamp2 colocalize extensively, we used time-lapse imaging of Vamp2-GFP as a proxy to characterize Kif1a-mediated vesicle transport in WT and Dcx/Dclk1 single and double deficient neurons, as well as following Dcx rescue (Figure 3, Movie S2). We found significantly fewer Vamp2-GFP vesicles exiting the cell body toward the neurites in both Dcx- and Dcx/Dclk1-deficient neurons (Figures 3A–3D); however, this effect can be rescued by overexpression of RNAi-resistant human Dcx (Figures 3D and 3E). Dcx/Dclk1-deficient neurons also show fewer Vamp2-GFP vesicles in neurites (Figure 3F). Extensive control experiments indicate that the transport defects caused by loss of Dcx/Dclk1 do not reflect changes in actin structure—including the “actin filter” (Song et al., 2009)—MT structure; common posttranslational modifications of MTs (Hammond et al., 2008, 2010) such as glutamylation, (de)tyrosination, and acetylation;, or alterations in other MAPs, such as MAP2 or tau-1 (data not shown).

**Figure 4:**
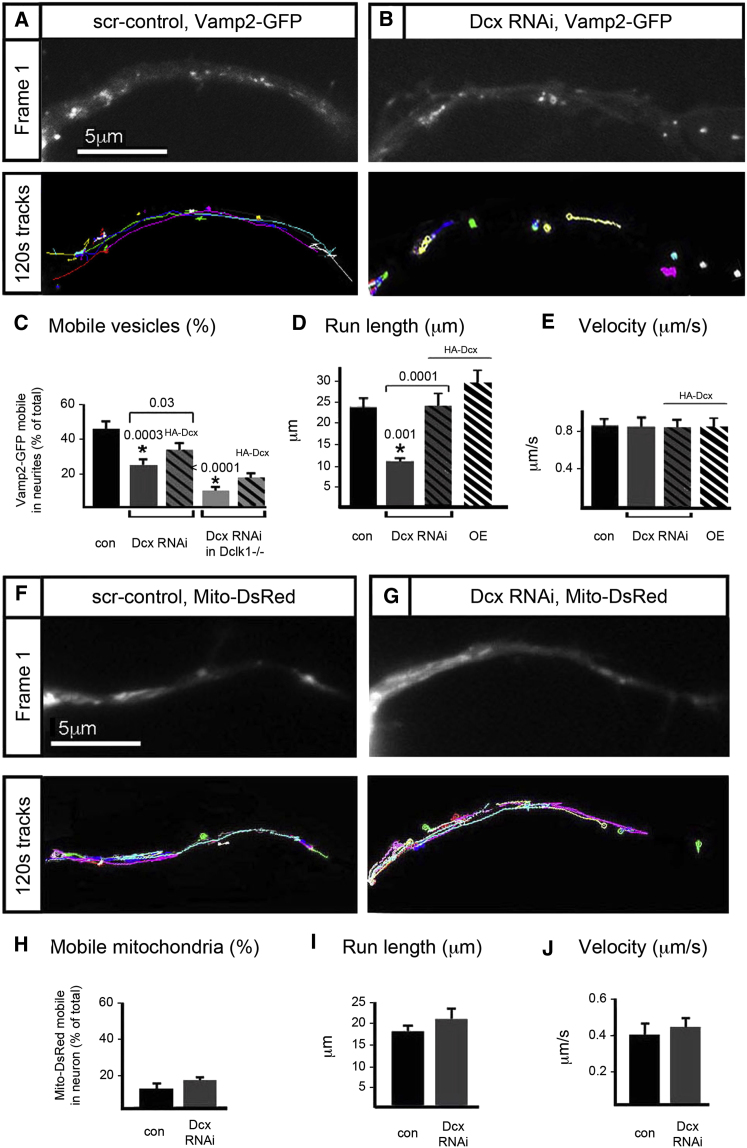
The Run Length of Vamp2-GFP in Dcx/Dclk1-Deficient Neurons Is Decreased (A–C) WT neurons treated with a scrambled control (A) and Dcx shRNAi (B) are then transfected with a plasmid for expression of Vamp2-GFP for live imaging. The top panel shows the first frame, and the bottom panel shows the tracks of Vamp2-GFP transport packets within the neurites. Each color represents the track of a single Vamp2 vesicle over the full 120 s. (C) Vamp2-GFP vesicles were analyzed for number of mobile vesicles in Dcx RNAi-treated neurons, Dcx/Dclk1 double deficient neurons, and rescue conditions. (D) Average run lengths are shown for each condition. This analysis excluded Vamp2-GFP vesicles that moved less than 3 μm in 120 s, as these may reflect vesicles in which the necessary components (e.g., MT, motor, cargo) are not properly complexed. (E) Velocity is shown in Dcx/ Dclk1-deficient, rescue, or overexpression conditions. (F–J) Mitochondrial transport is imaged in control neurons (F) and Dcx shRNAi neurons (G) using transfection with Mito-DsRed. The top panel shows the first frame, and the bottom panel shows the tracks of Mito-DsRed within the neurites over 120 s. Mitochondrial transport in neurites does not change significantly in terms of percent mobile organelles (H), run length (I), and velocity (J). Error bars in all panels represent the SEM. Scale bar, 5 μm in all panels.

Since endogenous Dcx is primarily localized to MTs in distal regions of the neurites, we sought to understand how Kif1a function changes distally in Dcx/Dclk1-deficient neurons by imaging and tracking Vamp2-GFP anterograde transport in distal neurites (Figures 4A–4E, Movie S3). In Dcx-deficient neurons, vesicle tracings were shorter than in neurons treated with a scrambled control shRNA. Quantification of vesicle behavior in both the single and double deficient Dcx/Dclk1 condition demonstrated statistically significant decreases in the number of mobile vesicles in the distal neurite, which can be rescued by expression of shRNAi-resistant human Dcx (Figure 4C). In addition, despite the paucity of mobile vesicles in Dcx-deficient neurons, we were able to determine anterograde run lengths for Vamp2 transport in a sufficient number of vesicles in these neurons to observe a significant decrease compared to WT (Figure 4D). On the other hand, the anterograde velocity of WT, Dcx RNAi, rescue, and Dcx overexpression does not change (Figure 4E). The highly abnormal Kif1a-mediated transport is all the more striking when compared to conventional kinesin function, which is unaffected in Dcx/Dclk1-deficient neurons (Figure 1, Figure S1). Cargo that is transported by conventional kinesin rather than Kif1a, such as mitochondria, was unaffected by Dcx deficiency, in terms of percentage of mobile mitochondria and run length between WT control and Dcx-deficient neurons (Figures 4F–4J, Movie S4). Consequently, we conclude that MTs in Dcx/Dclk1-deficient neurons are unimpaired in their ability to support motor-mediated transport related to conventional kinesin, but severely deficient for Kif1a, suggesting a kinesin subtype-specific role for the Dcx domain proteins Dcx/Dclk1 in the regulation of MT-based transport.

### Disease-Associated Dcx Mutations Disrupt Kif1a Function

**Figure 5:**
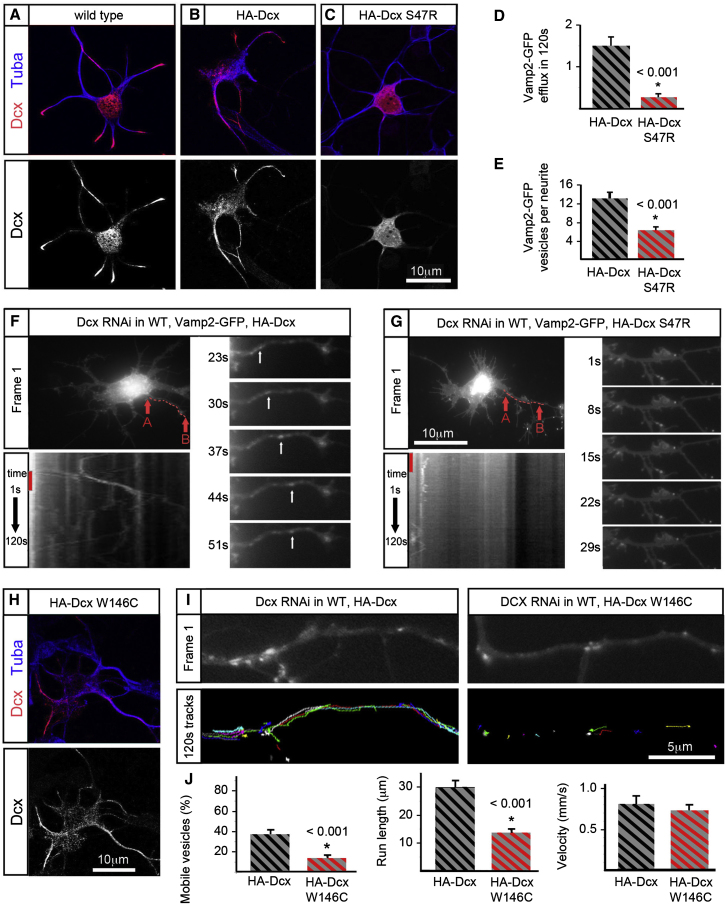
Causative Mutations for Lissencephaly Alter Kif1a/Vamp2 Transport (A) Dcx binding to MTs in normal neurons is shown. (B and C) Neurons are transfected with Dcx shRNAi and rescued with HA-tagged WT or mutant Dcx constructs resistant to the shRNAi. (B) depicts the distribution of WT HA-Dcx, which is similar to that of endogenous Dcx with more Dcx in the distal neurites, albeit higher levels of Dcx overall. (C) Mutant Dcx S47R binds only in the cell body. (D–G) Vamp2-GFP transport out of the cell body into neurites is shown in Dcx shRNAi neurons rescued by either WT HA-Dcx or HA-Dcx S47R. (D and E) Both efflux of Vamp2-GFP and number of Vamp2-GFP vesicles in neurites are shown for rescue with either WT HA-DcxS47R. (F and G) The top left panel is the first frame of the imaging study. A red, broken line 10 μm in length shows the region of the neurite used for generating the kymograph in the bottom left panel. The kymograph is created using the pixels selected by the tracing of the neurite from point A to point B. These pixels are aligned sequentially from the first frame to the last frame so that vesicle movement in the region of interest is shown throughout the imaging study. A red line on the left marks the 28 s time interval depicted by frames in the panel on the right. The right panels of (F) and (G) show frames of the neurite used to generate the kymograph at 7 s intervals. (H) The Dcx mutation W146C does not affect the MT binding of Dcx. (I) Top panels show the first frame of the time-lapse sequences used to generate the Vamp2-GFP tracks shown in the bottom panel for rescue with either WT HA-Dcx or HA-Dcx W146C. (J) Numbers of mobile vesicles, run length, and velocity are quantified in the WT HA-Dcx and HA-Dcx W146C rescue conditions. Error bars in all panels represent the SEM.

Since point mutations in Dcx are known to cause defects in neuronal migration, we tested whether some mutant Dcx proteins could potentially disrupt specific Kif1a transport functions. Dcx is known to decorate MTs in a gradient, with highest levels bound in a polarized manner in the cell body and in distal neurites (Figure 5A; Tint et al., 2009). We hypothesized that Dcx association with MTs may serve to prevent the Kif1a:cargo complex from dissociating from the MT, thus permitting longer run lengths along the MTs and resulting in efflux of the Kif1a:cargo complex from the soma into the neurites. We investigated the Dcx S47R mutation that causes retention of Dcx in the soma to determine its effect on Kif1a transport from the cell body into the neurite. *Dcx*^−*/y*^ neurons were transfected with plasmids expressing HA-tagged WT or mutant Dcx constructs, then permeabilized in MT-stabilizing buffer to determine where the exogenously expressed Dcx was bound in these neurons. *Dcx*^−*/y*^ neurons rescued with the WT HA-Dcx construct showed the greatest amount of Dcx in the distal regions of the neurites, as well as polarized binding in the soma (Figures 5A and 5B). In contrast, rescue with Dcx S47R results in retention of the mutant Dcx in the soma of the neuron without polarized Dcx binding in the cell body (Figure 5C). Live imaging of Vamp2-GFP to characterize Kif1a function in the same neurons rescued with HA-tagged Dcx S47R constructs demonstrates Dcx retention in the cell body and a significant reduction in the number of Vamp2-GFP vesicles exiting into neurites (Figures 5F and 5G). We conclude that Kif1a-dependent Vamp2 transport from the cell body into neurites is dependent on proper distribution of Dcx on MTs, i.e., polarized Dcx MT binding in the cell body and gradient in the neurites, suggesting that the critical interaction between Dcx and Kif1a is likely to occur at the MT surface.

We also examined human mutations in the sequence linking the N-DC and C-DC MT binding domains of Dcx. As the DC domains bind to MT recesses, we predict this “linker” sequence to be potentially exposed on the MT surface and interact with Kif1a. While MT binding of the Dcx W146C mutant appears to be intact in neurons, rescue of Dcx shRNAi-treated neurons with the Dcx W146C and K174E linker mutants did not rescue the dendritic polarity defects in neuronal morphology (Figures S3A–S3E). Live-cell imaging of Vamp2-GFP in Dcx shRNAi-treated neurons rescued with Dcx W146C shows a defect in vesicle transport (Figures 5H–5J, Movie S5). We tracked Vamp2-GFP vesicular transport in these neurites and found a decrease in the number of mobile vesicles compared with WT Dcx rescue neurons. Analysis of the mobile vesicles showed that run lengths were decreased in the Dcx W146C mutant rescue compared to WT, but that velocity was not significantly altered, remarkably similar to the effects of Dcx deficiency, suggesting that the interactions between Kif1a and Dcx may require specific amino acid residues in this linker segment.

### Dcx and Kif1a Form a Ternary Complex on the MT

**Figure 6:**
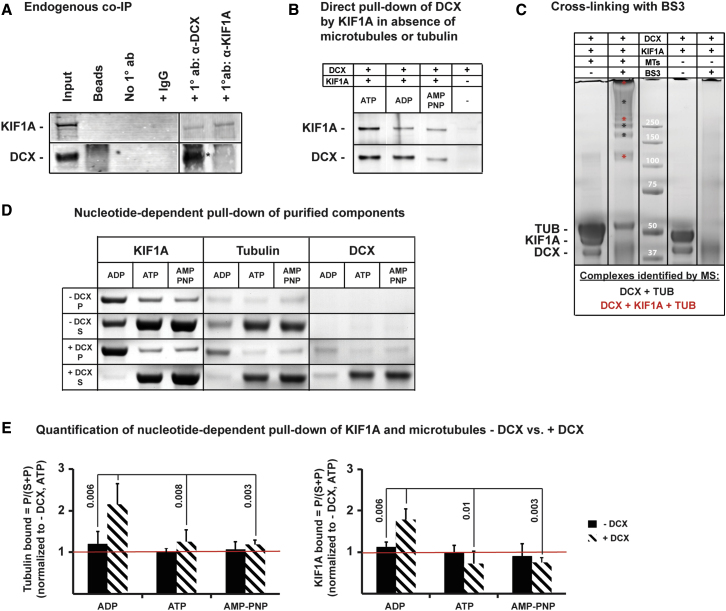
Dcx Interacts with Kif1a and Facilitates Binding of the Low-Affinity, ADP-Bound Kif1a Motor to MTs (A) Coimmunoprecipitation of endogenous Dcx and Kif1a from human fetal cortex (23 weeks) was performed with antisera to Dcx and Kif1a, respectively, in 2 mM AMP-PNP using BSA-blocked protein G beads. Protein complexes were analyzed by western blot. Lane 1 shows the original protein lysate at a 1:20 dilution. Lanes 2–4 are negative controls: (2) blocked protein G beads without lysate, (3) beads incubated with lysate but without antibody, (4) beads incubated with lysate and a nonspecific IgG antibody. Lane 5 shows pull-down of Kif1a with the primary polyclonal Dcx antibody; the Kif1a band is clearly visible. Lane 6 shows pull-down of Kif1a with the primary Kif1a antibody, but very little Dcx coimmunoprecipitates (faint band marked by asterisk). (B) Direct pull-down of overexpressed and purified full-length human Dcx by an N-terminal HaloTag human Kif1a (amino acids 1–361) fusion protein was performed in the presence of 4 mM nucleotides and 5 μM of each protein using HaloLink magnetic beads. Lanes 1–3 show that Dcx and the motor domain of Kif1a interact independently in the absence of MTs and the presence of either ATP, ADP, or AMP-PNP; the presence of excess Kif1a in the pull-down further suggests the existence of more than one binding site of the kinesin-3 motor domain on Dcx-decorated MTs. Lane 4 is a negative control. (C) Dcx and Kif1a form a ternary complex on the MT. Crosslinking was performed using BS3-d0 with purified human Dcx, Kif1a, and porcine MTs as shown. A range of crosslinked Dcx:MT:Kif1a complexes was identified as indicated by the red asterisks in lane 2. Crosslinking in the absence of MTs (lane 4) did not yield any visible bands. (D) Nucleotide-dependent pull-down of MTs in the presence and absence of full-length human Dcx by an N-terminal HaloTag human Kif1a (amino acids 1–361) fusion protein was performed in presence of 4 mM nucleotides and 5 μM of each protein component using HaloLink magnetic beads. Supernatant and pellet fractions are shown to indicate equal total protein loading for each nucleotide condition, and both fractions were used to quantify band intensities by densitometry after silver staining. (E) Quantification of (D) shows that Kif1a binding to MTs in the presence of Dcx and 4 μM nucleotide is significantly enhanced by addition of ADP, but not ATP or AMP-PNP (left panel) when compared to binding in absence of Dcx. Similarly, Dcx enhances pull-down of excess Kif1a motor domain in the ADP binding state, but not in the ATP or AMP-PNP binding state (right panel). Bound fractions were calculated as P/(S+P), and all quantifications are normalized to ATP in absence of DCX as indicated by the red line across all graphs. Error bars represent standard deviation, and significant p values are shown (two-tailed t test, n ≥ 3).

The changes in run length caused by alterations in Dcx levels in our mutational analysis suggested that Dcx might regulate Kif1a interactions with MTs. Coimmunoprecipitation (coIP) using a primary antiserum to the C terminus of Dcx on protein lysates of human fetal cortex (23 weeks of gestation) enriches for a complex that includes Kif1a, providing evidence of Dcx association in vivo (Figure 6A). This interaction was confirmed by the direct pull-down of Dcx using an N-terminal HaloTag human KIF1A (amino acids 1–361) fusion protein in the absence of MTs or tubulin using purified protein components (Figure 6B). Indeed, crosslinking experiments using bis(sulfosuccinimidyl)suberate (BS3) to reconstitute this complex using purified protein components in vitro followed by mass spectrometry analysis identified a range of molecular complexes containing both Dcx and Kif1a in the presence of MTs (Figure 6C). Interestingly, formation of the Dcx:Kif1a complex was also observed in the absence of MTs and nucleotide at concentrations >15 μM (data not shown). Therefore, our data suggest that Kif1a and Dcx can interact directly and independently of MTs, though when MTs are present that interaction likely occurs at the MT surface.

### Dcx Enhances the Affinity of the ADP-Bound Kif1a Motor for MTs

While we show that Dcx, Kif1a, and tubulin form a ternary complex both in vivo and in vitro, we asked whether Dcx has any effect on the direct interaction of Kif1a with MTs. Using a traditional MT pull-down assay and the nonhydrolyzable nucleotide AMP-PNP, which promotes high-affinity motor-MT interactions, lysates from Dcx/Dclk1-deficient mouse brain (*Dcx*^−*/y*^*;Dclk1*^*−/−*^*)* show significantly less Kif1a bound to MTs compared to WT (Figure S4A). Similarly, lysates from HEK cells expressing the motor domain of Kif1 (amino acids 1–365) and only the MT binding domain of Dcx (amino acids 1–270) show a modest, but significant, increase in binding of Kif1a to MTs in presence of excess MTs by 15%–20% over control (Figures S4B–S4D).

Because the effect of binding with AMP-PNP was relatively small in contrast to the run length effect observed in vivo, we assessed the effect of Dcx on the Kif1a-MT interaction (Nitta et al., 2004) in the presence of other nucleotides, i.e., ADP and ATP, using purified protein components. We bound purified N-terminally Halo-tagged, truncated human Kif1a (amino acids 1–361) to magnetic HaloLink beads under saturated conditions and coincubated it in the presence or absence of Dcx-decorated MTs and either ATP, ADP, or AMP-PNP (Figures 6D and 6E). Strikingly, while Dcx does not appear to enhance motor binding to MTs in the ATP and AMP-PNP binding state, a significant increase is observed in the ADP binding state when Dcx is present. The Dcx-mediated increase in the presence of ADP is approximately 2-fold compared to a Dcx-negative control and compared to ATP or AMP-PNP in the presence of Dcx. Interestingly, since the experiment is performed under saturating conditions, excess Kif1a protein is pulled down in the presence of Dcx and ADP by a factor of 2-fold over ATP and AMP-PNP and/or lack of Dcx (Figure 6E, right panel). This suggests the possible existence of two binding sites on Dcx, one that is nucleotide independent and one that is specific for ADP-bound Kif1a. Similar results are observed when performing the reverse experiment, where human Dcx is bound to the magnetic beads first, followed by coincubation with Kif1a (C351) in the presence of all other components (data not shown), thus confirming our results shown in Figure 6D.

### Molecular Basis of Dcx-Kinesin Crosstalk on the MT Surface

**Figure 7:**
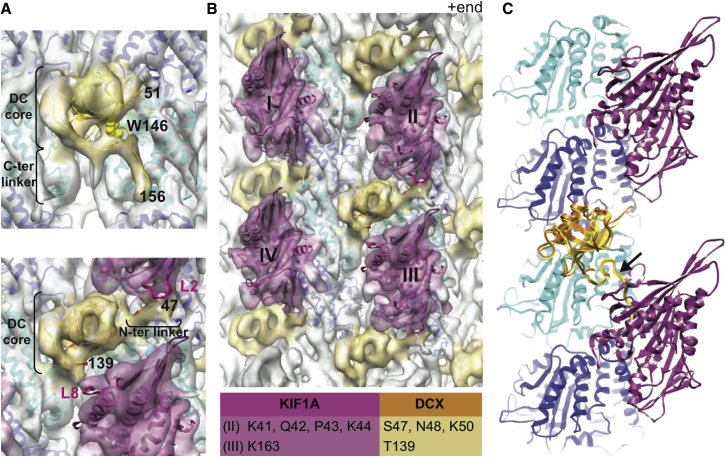
Model of Dcx MT Binding in the Presence and Absence of Kif1a (A) Cryo-EM structures of Dcx-MTs alone (top panel, 8.3 Å resolution, see Figure S5A) and in the presence of the kinesin motor domain (bottom panel; Fourniol et al., 2010); transparent surface, tubulin colored in gray, kinesin in faded pink, Dcx in yellow, docked with atomic coordinates (ribbons) of tubulin (2XRP.PDB, alpha in blue, beta in cyan, crosscorrelation of the fit; top panel, 0.720; bottom panel, 0.744). Kinesin binding affects the structure/flexibility of the linker regions N and C terminal of N-DC (residues at boundaries are numbered). Kif1a loops L2 and L8 (bright pink) are likely in direct contact with linker regions. In the absence of kinesin (N-DC colored yellow), extra density in the reconstruction enabled modeling of the C-terminal linker docked against N-DC through W146 and in good agreement with NMR studies (crosscorrelation 0.684 before and 0.739 after modeling; Kim et al., 2003; also see Figure S5B). The plus end of the MT is oriented upward. (B) Resolution 8.2 Å cryo-electron microscopy reconstruction of Dcx-MTs codecorated with conventional kinesin motor domain (Fourniol et al., 2010; also see Figure S5C), Dcx R1 (1MJD.PDB, model 11, amino acids 46–139, orange, crosscorrelation 0.722) and KIF1A (1I5S.PDB, dark pink, crosscorrelation 0.711). The bound DC domain is surrounded by four motor domains (labeled I–IV), and is <5 Å from motor domains II and III: residues in Kif1A and N-DC separated by less than 5 Å are listed in the table. (C) The superimposition of pseudoatomic models of MT-bound Dcx in the presence (orange ribbon) and absence (yellow ribbon) of Kif1a motor domain (dark pink) shows a clash between the C-terminal linker of N-DC (arrow, thicker yellow ribbon) and Kif1a, explaining why that linker is induced to undock upon motor binding (also see Movie S6).

To investigate the molecular basis of our cellular and biophysical observations, we first examined whether the conformation of MT-bound Dcx is influenced by the absence or presence of kinesin. In a subnanometer-resolution cryo-EM reconstruction of the binary Dcx:MT complex, we clearly observed a DC core at the Dcx binding site (Figure 7A, top; Figure S5A). This was also previously observed in a reconstruction of a ternary Dcx:MT:kinesin complex (Figure 7A, bottom; Fourniol et al., 2010), but due to technical limitations in our reconstruction method, this could have corresponded to N-DC, C-DC, or a mixture of both. Strikingly, in our new structure we found that linker regions on either side of the well-defined DC core adopt a significantly different conformation in the absence compared to the presence of bound kinesin, providing important insight into the Dcx-MT interaction (Figure 7A).

In the Dcx:MT binary complex (Figure 7A, top), the pre-DC linker region shows only diffuse density, demonstrating that this region is flexible when bound to MTs, as it is in solution (Kim et al., 2003; Figure S5B). Crucially, in the absence of kinesin, there is clearly post-DC linker density docking along the DC core (Figure 7A, top). A distinctive feature of N-DC is the presence of W146 in its post-N-DC linker, which has been shown to dynamically dock against the N-DC core (Cierpicki et al., 2006); point mutations at this residue cause lissencephaly (Leger et al., 2008) and defects in intracellular transport (Figure 5). An equivalent hydrophobic residue is not present in the post-C-DC linker, nor is the linker seen docked against the C-DC in its solution structure (PDB ID code 2DNF; Figure S5B). Flexible docking of available N/C-DC structures—including the post-DC linker—into our cryo-EM reconstruction confirmed that W146 apparently contributes to docking of the extra density against the DC core when bound to MTs. This observation strongly supports the idea that the Dcx density observed in our reconstructions corresponds to N-DC.

Comparison of the Dcx:MT and DCX:MT:kinesin complexes also provided significant insight into the nature of the contacts between Dcx and kinesin on the MT surface. In the Dcx:MT:kinesin ternary complex (Figure 7B; Fourniol et al., 2010), although Dcx binds at the corner of four tubulin dimers, and therefore four kinesin motor domains (MDs I–IV), the Dcx density is more closely associated with kinesin motors along one of the protofilaments (Figure 7B, MDs II and III). In this reconstruction, the MD is in a high-affinity nucleotide-free state and enabled docking of a Kif1a MD crystal structure (Figure 7B, Figure S5C). Residues in kinesin_II_ (loop 2) and kinesin_III_ (loop 8)—which may be important for axonal specificity of some kinesins (Huang and Banker, 2011)—but not kinesin_I_ or kinesin_IV_, are closer than 5Å to the Dcx density (Figures 7A and B). Intriguingly, the N-terminal linker region outside the proposed N-DC core interacts with the MT wall and lies close to kinesin_II_ loop 2 (Figure 7A), while the C-terminal linker is completely displaced from N-DC due to the presence of the bound motor (kinesin_III_). Thus, because their conformation is significantly different in the absence and presence of motor protein, it is likely that residues in the linkers adjacent to the N-DC domain interact with kinesin at the MT surface (Figure 7B, table) and dynamically respond to the presence or absence of bound motor (Figure 7C). By its nature of loose attachment, the motor's low-affinity ADP-bound state is hard to access by subnanometer-resolution cryo-EM structure determination. However, our structural analysis suggests specific residues on Dcx and Kif1a that could also act selectively to enhance the binding of the low-affinity ADP-bound motor for MTs (Figure S5D). Although not visible in our reconstruction due to its flexibility, C-terminal portions of Dcx could also be involved in this interaction.

## Discussion

Here, we show that the Dcx domain proteins Dcx and Dclk1 regulate the function of the neuronal kinesin-3 Kif1a. Dcx- and/or Dclk1-deficient neurons show impaired Kif1a-mediated transport of Vamp2. Lack of Dcx and/or Dclk1 decreases run length of the motor protein and its associated cargo. We show that these changes in motor behavior are correlated with enhanced binding of the ADP-bound Kif1a motor domain to MTs in the presence of Dcx. In addition, we show that mutations in the linker region of Dcx impair Kif1a motility. Finally, using cryo-EM and subnanometer-resolution reconstruction, we visualize the kinesin-dependent conformational variability of the pre- and post-N-DC linker region of MT-bound Dcx, which likely contributes to regulation of motor function.

### Regulation of Kif1a Motor Domain Function through Interactions with Dcx

Dcx/Dclk1 regulation at the MT surface represents a mechanism of Kif1a regulation—distinct from cargo binding and release of autoinhibition, dimerization, or interactions with polyglutamylated tubulins (Verhey and Hammond, 2009). Instead, our data suggest that a MAP, in this case Dcx, can enhance motor function by increasing run length. This increase in run length correlates with an approximately 2-fold increase in affinity of the ADP-bound Kif1a motor domain to MTs in the presence of Dcx, suggesting that fine regulation of the weak affinity state of Kif1a can titrate motor activity locally in critical regions of the neuron where Dcx is enriched on MTs. Our findings suggest that Dcx may regulate run length of kinesin-3 motors through specific reduction of the “off-rate” kinetics of the motor protein, thus reducing the likelihood that the motor domain detaches from the MT after completion of its ATPase cycle that drives processive movement along the protofilament. Differential binding of Dcx and/or Dclk1 to the MT, due to local concentration and/or posttranslational modification of Dcx/Dclk1 (Bielas et al., 2007; Bilimoria et al., 2010; Gdalyahu et al., 2004; Tanaka et al., 2004), then has the potential to provide local control of Kif1a-mediated vesicle trafficking.

### Structural Analysis of the Molecular Basis of the Dcx-Kinesin Interaction on the MT

The comparative analysis of the Dcx:MT complex versus the Dcx:MT:kinesin complex allows us to identify residues that might be involved in the specific regulation of the Dcx-Kif1a interaction (Figure 7B, table). Our data show that the region of Dcx bound to the MT corresponds to the N-DC (R1) domain. Interestingly, in the absence of motor binding to the MT, the post-N-DC linker region of Dcx is clearly docked on the MT through W146, which is consistent with previously reported results (Cierpicki et al., 2006). Addition of a kinesin MD, however, results in the undocking of the post-N-DC linker and, in addition, orientation of the pre-N-DC density in close proximity between two kinesin motor domains (Figure 7A, bottom; and Figure 7B). Taken together with our live-cell imaging data reported for the human patient mutations W146C and S47R in Dcx (Figure 5), which show both a reduced overall mobility of presynaptic vesicles and a decrease in run length, our structural analysis suggests a crucial role of the N-DC domain of Dcx for regulating access of the Kif1a motor domain to MTs. It is likely that residues adjacent to the N-DC domain of Dcx interact with loop 2 and loop 8 of Kif1a at the MT surface (Figure 7B, table), thus allowing a dynamic response of Dcx to the presence or absence of the motor protein (Figure 7C, Movie S6). Although ultimately ternary reconstructions with Dcx and Kif1a will be necessary to reveal the structural and functional details of the MAP-motor interactions in the presence of different nucleotides, our current analysis yields a first interpretation of how Dcx could act selectively to enhance the binding of the low-affinity ADP-bound motor for MTs. In addition, the docking and undocking of the post-N-DC linker may be a mechanism for maintaining specificity of Kif1a binding to certain MTs that are decorated with Dcx, and thus perhaps excluding trafficking of cargo carried by other molecular motors not regulated by Dcx.

### Dynamic Spatial and Temporal Regulation of Vesicle Trafficking via Regulation of Dcx MT Binding: Implications for Dcx/Dclk1 in Neuronal Migration and Axonal Outgrowth

Dcx MT binding can be mediated by dephosphorylation of Dcx through a specific interaction with spinophilin and protein phosphatase 1 in distal regions of developing neurites (Bielas et al., 2007). The binding of Dcx on MTs in these regions may therefore dynamically regulate vesicle trafficking in these regions, potentially allowing local mechanisms at the growth cone to regulate trafficking into the growth cone. Spinophilin itself is an actin-binding protein with a role in spine formation (Feng et al., 2000). Thus, positive signals directing Dcx MT binding may be downstream of actin effects in spines or dendrites and may stimulate Kif1a transport potentially in a dynamic and localized fashion in both dendrites as well as axons. In contrast, release of Dcx from MTs is mediated by kinases that include many with important roles in neuronal development and function, such as CDK5, MARK, and JunK (Chae et al., 1997; Gdalyahu et al., 2004; Gilmore et al., 1998; Tanaka et al., 2004). Therefore, spatial and temporal regulation of Dcx phosphorylation and dephosphorylation presents a potential means of regulating Kif1a transport in a local fashion.

The interaction between Kif1a and Dcx/Dclk1 for locally regulating transport suggests mechanisms for the role of Dcx/Dclk1 in neuronal migration and axonal outgrowth (Deuel et al., 2006; Friocourt et al., 2011). Neurons undergo changes in shape during migration which require dynamic regulation of trafficking and membrane remodeling in response to extracellular signals. Newly born neuroblasts are initially bipolar but become multipolar in the subventricular zone before becoming bipolar migrating neurons that undergo saltatory movements of the leading process and the nucleus as they traverse the intermediate zone and CP (Nadarajah and Parnavelas, 2002; Noctor et al., 2004; Solecki et al., 2006). Our data (Figure S3 and data not shown), as well as previously published studies (Bai et al., 2003; Koizumi et al., 2006), show that Kif1a and Dcx/Dclk1 are clearly essential for remodeling the multipolar shape into a bipolar shape, which may reflect roles in dynamic regulation of membrane remodeling through changes in vesicle trafficking, though Kif1a-based motor function may also have other roles in migratory mechanisms (Tsai et al., 2010).

### A General Role for Doublecortin Family Proteins

Dcx and Dclk1 are part of an enlarging family of Dcx domain-containing MAPs that all appear to localize to MTs (Coquelle et al., 2006; Reiner et al., 2006). The widespread expression of Dcx domain proteins suggests a broader role of this unique family of MAPs in neuronal function. While Dcx is a developmentally expressed protein, Dclk1 is present in both immature and adult neurons. Though the Kif1a-binding linker sequence is highly conserved between Dcx and Dclk1, and appears to regulate Kif1a but not conventional kinesin, this linker sequence is highly divergent in sequence in other Dcx domain-containing proteins such as RP1 and DCDC2 (Figure S3A). This lack of conservation in a potential kinesin-binding interface suggests a model in which DC domain proteins interact with kinesins or other motors in combinatorial fashion, potentially even forming a broader “MAP-kinesin code,” in which MAPs and kinesins show diverse yet specific interactions. Other MAPs bind MTs at various sites and with structurally diverse MT-binding domains, and it is an open question whether non-Dcx domain-containing MAPs may regulate more structurally diverse kinesin family members. Although we have by no means explored the full diversity or specificity of interactions between MAPs and kinesins, our data provide an initial step that may ultimately enlighten the great diversity of these large protein classes.

## Experimental Procedures

Detailed methods can be found in the Supplemental Information.

### Generation of Dcx^−/y^;*Dclk1*^−/−^ Mutant Mice

Animal work was conducted in compliance with protocols approved by the Children's National Medical Center Institutional Animal Care and Use Committee. We bred *Dcx*^+/−^;*Dclk1*^+/−^ females with *Dclk1*^−/−^ males to generate Dcx^−/y^;*Dclk1*^−/−^ mice as described previously (Deuel et al., 2006).

### Dissociation and Transfection of Neurons

E15 cortices or E17.5 hippocampi were dissected and dissociated using the Worthington Papain dissociation system. Plasmids were transfected by using the Amaxa system of electroporation at the time of dissociation according to instructions from the mouse neuronal transfection protocol or with Lipofectamine 2000 overnight prior to imaging. Details of DNA constructs are in the Supplemental Experimental Procedures.

### Confocal Microscopy and Data Analysis

Images were acquired using a Zeiss LSM510 confocal microscope. The measurement of pixel intensity in the neurite as a function of distance from the cell body was conducted using MetaMorph. For the tracing and measurement of neurites using the ImageJ plug-in NeuronJ, 20 randomly selected neurons were measured in each category from more than five representative experiments.

### Time-Lapse Imaging

E17.5 neurons were transfected with shRNAi constructs by the standard Amaxa transfection protocol after dissociation and plating. They were again transfected with either Lipofectamine 2000 with VAMP-2 or Mito-DsRed 18 hr prior to imaging, which commenced 96 hr after initial transfection under controlled atmospheric conditions at 37°C. Images were acquired on an inverted epifluorescence microscope (IX-81, Olympus America Inc., Melville, NY) equipped with high numerical aperture lenses (Apo 60× NA 1.49, Olympus) and a stage top incubator (Tokaihit, Japan) maintained at 37°C at a rate of one capture per second for the VAMP2 and one capture every 2 s for the mitoDsRed. Fluorescence excitation was carried out using solid-state lasers (Melles Griot, Carlsbad, CA) emitting at 488 nm (for green) and 561 nm (for red) fluorophores. Emission was collected through appropriate emission band-pass filters obtained from Chroma Technologies Corp. (Brattleboro, VT). Images were acquired with a 12-bit cooled CCD ORCA-ER (Hamamatsu Photonics) with a resolution of 1280 × 1024 pixels (pixel size = 6.45 μm^2^). The camera, lasers, and shutters were all controlled using Slidebook 5 (Intelligent Imaging Innovations, Denver, CO). Analysis of time-lapse imaging was performed with MetaMorph for tracking and the ImageJ Manual Tracking plugin as described (http://rsbweb.nih.gov/ij/plugins/track/track.html).

### Crosslinking Experiments

Crosslinking experiments were performed using a 1:1 mixture of BS3-d0 and BS3-d4 (Pierce). For each reaction, 10 μM of each purified protein component (Dcx, Kif1a, MTs) and 4 mM ATP were incubated in a 25 μl reaction in BRB80 buffer (80 mM PIPES, 1 mM EGTA, 1 mM MgCl2 [pH 6.9]) for 30–45 min at room temperature (rocking) to allow ternary complex formation. After this initial incubation period, the crosslinking reagent BS3 was added in 100-fold excess (1 mM), and the reaction was incubated at room temperature for another 60 min (rocking). Reactions in which MTs and/or BS3 were omitted were carried out exactly as described above, but lacking the indicated components. The results were assessed by SDS-PAGE and silver staining, followed by protein identification by mass spectrometry.

### Nucleotide-Dependent Pull-Down of Purified Components

Nucleotide-dependent coimmunoprecipitation of Dcx and Kif1a was performed using purified Halo-tagged human Dcx and Kif1a protein (as described above) and HaloLink Magnetic Beads (Promega). MTs and unpolymerized porcine tubulin were purchased from Cytoskeleton. For each reaction, 4–10 μM of each purified protein component (Dcx, Kif1a, MTs, or tubulin) and 4 mM nucleotide (ATP, ADP, AMP-PNP) were incubated in a 50 μl reaction in HaloLink binding/washing buffer (100 mM Tris [pH 7.4], 150 mM NaCl, 4 mM MgCl_2_, 1 mM EGTA, 1 mM DTT, 0.5% Triton X-100) for 15–45 min at room temperature (rocking) to allow ternary complex formation. After this initial incubation period, 25 μl of BSA-blocked magnetic beads were added to each reaction, followed by further incubation at room temperature for another 15–45 min (rocking). Reactions in which MTs, tubulin, and/or Dcx or Kif1a were omitted were carried out exactly as described above, but lacking the indicated components. Each reaction was washed three times with HaloLink binding/washing buffer, and after addition of SDS-PAGE sample buffer, proteins were eluted off the beads by boiling the samples at 95°C for 3–5 min. The results were assessed by SDS-PAGE and silver staining, followed by densitometry analysis and quantification. Results shown in Figure 6I were quantified by normalizing the bound amount of Kif1a or tubulin as p = P/(S+P) to the ATP condition in absence of Dcx.

### Cryo-EM and Image Reconstruction of Dcx-MTs

Recombinant full-length human Dcx (amino acids 1–366) was expressed and purified from Sf9 insect cells, and Dcx-stabilized MT cryo-EM samples were prepared as described previously (Fourniol et al., 2010; Moores et al., 2004), except that no kinesin motor domain was added. Low-dose images were collected on a FEG electron microscope (Tecnai F20) operating at 200 kV, 50,000× magnification and with a defocus of 0.9–3.6 μm. Micrographs were recorded on films (Kodak SO-163) and binned to final sampling of 2.8 Å/pixel. MTs (222) were selected and processed following a single particle procedure described elsewhere (Fourniol et al., 2010; Sindelar and Downing, 2010). A subselection of 146,000 tubulin dimers (75% of total data set) went into the final reconstruction. The resolution of 8.3 Å was assessed using the Fourier shell correlation (FSC) 0.5 criterion, calculated between two reconstructions from independent half data sets (see Figure S7B). EM maps are deposited in the EMDB: Dcx-MT EMDB ID 2095 and Dcx-MT-kinesin EMDB ID 2098.

### Pseudoatomic Model Building

UCSF Chimera (Pettersen et al., 2004) was used for visualization of 3D models, atomic model building, and rigid-body fitting of atomic models in the cryo-EM density maps. The N-DC domain atomic model (1MJD.pdb, model 11, amino acids 38–150) was extended at its C terminus (amino acids 151–156). The resolution of our reconstruction is not sufficiently high to unambiguously determine the conformation of the extended C-terminal linker. Nevertheless, we modeled an extended polypeptide chain into the Dcx cryo-EM density to evaluate its approximate position on the MT surface and with respect to the kinesin binding site. Briefly, the Dcx cryo-EM density was isolated, the extended N-DC domain docked into it, and the C-terminal polypeptide chain was moved into the EM density using flexible fitting with Flex-EM (the core DC domain, amino acids 53–129; and the docked W146 region, amino acids 145–146, were treated as a single rigid body; Topf et al., 2008). Finally multiple subunit fitting (extended N-DC domain with four surrounding tubulin monomers) was performed using Flex-EM. The kinesin neck-linker (amino acids 324–329) was modeled into the Dcx:MT:kinesin cryo-EM map in a similar way. Because of apparent local displacements between 1BG2.PDB and the kinesin density seen in our reconstruction, we refined the position of loop 2, loop 9, and helix α6 using Flex-EM, but the larger conformational changes of loop 11 could not be modeled simply; also seen by Sindelar and Downing (2010).

## Accession Numbers

Reconstructions were deposited in the Electron Microscopy Data Bank (EMDB) under the following accession numbers: 2095 (Dcx-MT reconstruction) and 2098 (Dcx-MT-kinesin reconstruction). All pseudoatomic models are deposited in the Protein Data Bank (PDB): Dcx-MT PDB ID code 4ATU and Dcx-MT-kinesin PDB ID code 4ATX.

## Supplemental Information

Document S1. Supplemental Experimental Procedures, Figure S1, Figure S2, Figure S3, Figure S4, Figure S5, and Supplemental References

Movie S1. Transport of Vamp2-GFP in Kif1a RNAi-Treated Neurons On dissociation, neurons are treated with a scrambled control, Kif1a shRNAi, or Kif1a shRNAi and myc-tagged human Kif1a (rescue). Neurons are transfected with Vamp2-GFP at 3 DIV and imaged at 4 DIV. Frames are acquired at one frame per second for 120 s. (Related to Figure 2.)

Movie S2. Efflux of Vamp2-GFP from the Cell Body into Neurites of Dcx/Dclk1-Deficient Neurons At the time of dissociation, wild type or *Dclk1*^−/−^ neurons are transfected with a scrambled control, Dcx shRNAi, or the Dcx shRNAi and an HA-tagged, RNAi-resistant Dcx (rescue). Neurons are transfected with Vamp2-GFP at 3 DIV and imaged at 4 DIV. Frames are acquired at one frame per second for 120 s. (Related to Figure 3.)

Movie S3. Transport of Vamp2-GFP in Distal Neurites of Dcx-Deficient Neurons Wild-type neurons are transfected with a scrambled control or an shRNAi Dcx construct at the time of dissociation. Plated neurons are transfected with Vamp2-GFP at 3 DIV and imaged at 4 DIV. Frames are acquired at one frame per second for 120 s. (Related to Figure 4.)

Movie S4. Transport of Mito-RFP in Neurites of Dcx-Deficient Neurons Wild-type neurons are transfected with a scrambled control or an shRNAi Dcx construct at the time of dissociation. Plated neurons are transfected with Mito-DsRed at 4 DIV and imaged at 5 DIV. Frames are acquired at one frame per second for 120 s. (Related to Figure 4.)

Movie S5. Transport of Vamp2-GFP in Dcx RNAi-Treated Neurons Rescued with HA-Dcx W146C Wild-type neurons are transfected with Dcx shRNAi and a HA-tagged Dcx rescue construct or an HA-tagged Dcx W146C mutant construct. Neurons are transfected with Vamp2-GFP at 3 DIV and imaged at 4 DIV. Frames are acquired at one frame per second for 120 s. (Related to Figure 5.)

Movie S6. Models of Dcx Binding to Microtubules in the Presence and Absence of Kinesin Highlight Conformational Rearrangements in the Bound Dcx Cryo-EM maps, fitted atomic coordinates, and rendering parameters as in Figure 7.

## References

[bib1] Bai J., Ramos R.L., Ackman J.B., Thomas A.M., Lee R.V., LoTurco J.J. (2003). RNAi reveals doublecortin is required for radial migration in rat neocortex. Nat. Neurosci..

[bib2] Bielas S.L., Serneo F.F., Chechlacz M., Deerinck T.J., Perkins G.A., Allen P.B., Ellisman M.H., Gleeson J.G. (2007). Spinophilin facilitates dephosphorylation of doublecortin by PP1 to mediate microtubule bundling at the axonal wrist. Cell.

[bib3] Bilimoria P.M., de la Torre-Ubieta L., Ikeuchi Y., Becker E.B., Reiner O., Bonni A. (2010). A JIP3-regulated GSK3beta/DCX signaling pathway restricts axon branching. J. Neurosci..

[bib4] Binder L.I., Frankfurter A., Rebhun L.I. (1986). Differential localization of MAP-2 and tau in mammalian neurons in situ. Ann. N Y Acad. Sci..

[bib5] Caviston J.P., Holzbaur E.L. (2006). Microtubule motors at the intersection of trafficking and transport. Trends Cell Biol..

[bib6] Chae T., Kwon Y.T., Bronson R., Dikkes P., Li E., Tsai L.H. (1997). Mice lacking p35, a neuronal specific activator of Cdk5, display cortical lamination defects, seizures, and adult lethality. Neuron.

[bib7] Cho K.I., Cai Y., Yi H., Yeh A., Aslanukov A., Ferreira P.A. (2007). Association of the kinesin-binding domain of RanBP2 to KIF5B and KIF5C determines mitochondria localization and function. Traffic.

[bib8] Cierpicki T., Kim M.H., Cooper D.R., Derewenda U., Bushweller J.H., Derewenda Z.S. (2006). The DC-module of doublecortin: dynamics, domain boundaries, and functional implications. Proteins.

[bib9] Coquelle F.M., Levy T., Bergmann S., Wolf S.G., Bar-El D., Sapir T., Brody Y., Orr I., Barkai N., Eichele G. (2006). Common and divergent roles for members of the mouse DCX superfamily. Cell Cycle.

[bib10] Dehmelt L., Halpain S. (2005). The MAP2/Tau family of microtubule-associated proteins. Genome Biol..

[bib11] des Portes V., Francis F., Pinard J.M., Desguerre I., Moutard M.L., Snoeck I., Meiners L.C., Capron F., Cusmai R., Ricci S. (1998). Doublecortin is the major gene causing X-linked subcortical laminar heterotopia (SCLH). Hum. Mol. Genet..

[bib12] Deuel T.A., Liu J.S., Corbo J.C., Yoo S.Y., Rorke-Adams L.B., Walsh C.A. (2006). Genetic interactions between doublecortin and doublecortin-like kinase in neuronal migration and axon outgrowth. Neuron.

[bib13] Duncan J.E., Goldstein L.S. (2006). The genetics of axonal transport and axonal transport disorders. PLoS Genet..

[bib14] Feng J., Yan Z., Ferreira A., Tomizawa K., Liauw J.A., Zhuo M., Allen P.B., Ouimet C.C., Greengard P. (2000). Spinophilin regulates the formation and function of dendritic spines. Proc. Natl. Acad. Sci. USA.

[bib15] Fourniol F.J., Sindelar C.V., Amigues B., Clare D.K., Thomas G., Perderiset M., Francis F., Houdusse A., Moores C.A. (2010). Template-free 13-protofilament microtubule-MAP assembly visualized at 8 A resolution. J. Cell Biol..

[bib16] Friocourt G., Marcorelles P., Saugier-Veber P., Quille M.L., Marret S., Laquerriere A. (2011). Role of cytoskeletal abnormalities in the neuropathology and pathophysiology of type I lissencephaly. Acta Neuropathol..

[bib17] Gdalyahu A., Ghosh I., Levy T., Sapir T., Sapoznik S., Fishler Y., Azoulai D., Reiner O. (2004). DCX, a new mediator of the JNK pathway. EMBO J..

[bib18] Gilmore E.C., Ohshima T., Goffinet A.M., Kulkarni A.B., Herrup K. (1998). Cyclin-dependent kinase 5-deficient mice demonstrate novel developmental arrest in cerebral cortex. J. Neurosci..

[bib19] Glater E.E., Megeath L.J., Stowers R.S., Schwarz T.L. (2006). Axonal transport of mitochondria requires milton to recruit kinesin heavy chain and is light chain independent. J. Cell Biol..

[bib20] Gleeson J.G., Allen K.M., Fox J.W., Lamperti E.D., Berkovic S., Scheffer I., Cooper E.C., Dobyns W.B., Minnerath S.R., Ross M.E. (1998). Doublecortin, a brain-specific gene mutated in human X-linked lissencephaly and double cortex syndrome, encodes a putative signaling protein. Cell.

[bib21] Gleeson J.G., Lin P.T., Flanagan L.A., Walsh C.A. (1999). Doublecortin is a microtubule-associated protein and is expressed widely by migrating neurons. Neuron.

[bib22] Hammond J.W., Cai D., Verhey K.J. (2008). Tubulin modifications and their cellular functions. Curr. Opin. Cell Biol..

[bib23] Hammond J.W., Cai D., Blasius T.L., Li Z., Jiang Y., Jih G.T., Meyhofer E., Verhey K.J. (2009). Mammalian Kinesin-3 motors are dimeric in vivo and move by processive motility upon release of autoinhibition. PLoS Biol..

[bib24] Hammond J.W., Huang C.F., Kaech S., Jacobson C., Banker G., Verhey K.J. (2010). Posttranslational modifications of tubulin and the polarized transport of kinesin-1 in neurons. Mol. Biol. Cell.

[bib25] Horesh D., Sapir T., Francis F., Wolf S.G., Caspi M., Elbaum M., Chelly J., Reiner O. (1999). Doublecortin, a stabilizer of microtubules. Hum. Mol. Genet..

[bib26] Huang C.F., Banker G. (2011). The translocation selectivity of the kinesins that mediate neuronal organelle transport. Traffic.

[bib27] Jacobson C., Schnapp B., Banker G.A. (2006). A change in the selective translocation of the Kinesin-1 motor domain marks the initial specification of the axon. Neuron.

[bib28] Kim M.H., Cierpicki T., Derewenda U., Krowarsch D., Feng Y., Devedjiev Y., Dauter Z., Walsh C.A., Otlewski J., Bushweller J.H. (2003). The DCX-domain tandems of doublecortin and doublecortin-like kinase. Nat. Struct. Biol..

[bib29] Koizumi H., Tanaka T., Gleeson J.G. (2006). Doublecortin-like kinase functions with doublecortin to mediate fiber tract decussation and neuronal migration. Neuron.

[bib30] Leger P.L., Souville I., Boddaert N., Elie C., Pinard J.M., Plouin P., Moutard M.L., des Portes V., Van Esch H., Joriot S. (2008). The location of DCX mutations predicts malformation severity in X-linked lissencephaly. Neurogenetics.

[bib31] Lin P.T., Gleeson J.G., Corbo J.C., Flanagan L., Walsh C.A. (2000). DCAMKL1 encodes a protein kinase with homology to doublecortin that regulates microtubule polymerization. J. Neurosci..

[bib32] Lo K.Y., Kuzmin A., Unger S.M., Petersen J.D., Silverman M.A. (2011). KIF1A is the primary anterograde motor protein required for the axonal transport of dense-core vesicles in cultured hippocampal neurons. Neurosci. Lett..

[bib33] Moores C.A., Perderiset M., Francis F., Chelly J., Houdusse A., Milligan R.A. (2004). Mechanism of microtubule stabilization by doublecortin. Mol. Cell.

[bib34] Nadarajah B., Parnavelas J.G. (2002). Modes of neuronal migration in the developing cerebral cortex. Nat. Rev. Neurosci..

[bib35] Nangaku M., Sato-Yoshitake R., Okada Y., Noda Y., Takemura R., Yamazaki H., Hirokawa N. (1994). KIF1B, a novel microtubule plus end-directed monomeric motor protein for transport of mitochondria. Cell.

[bib36] Nitta R., Kikkawa M., Okada Y., Hirokawa N. (2004). KIF1A alternately uses two loops to bind microtubules. Science.

[bib37] Noctor S.C., Martinez-Cerdeno V., Ivic L., Kriegstein A.R. (2004). Cortical neurons arise in symmetric and asymmetric division zones and migrate through specific phases. Nat. Neurosci..

[bib38] Okada Y., Hirokawa N. (1999). A processive single-headed motor: kinesin superfamily protein KIF1A. Science.

[bib39] Pettersen E.F., Goddard T.D., Huang C.C., Couch G.S., Greenblatt D.M., Meng E.C., Ferrin T.E. (2004). UCSF Chimera—a visualization system for exploratory research and analysis. J. Comput. Chem..

[bib40] Reiner O., Coquelle F.M., Peter B., Levy T., Kaplan A., Sapir T., Orr I., Barkai N., Eichele G., Bergmann S. (2006). The evolving doublecortin (DCX) superfamily. BMC Genomics.

[bib41] Sapir T., Horesh D., Caspi M., Atlas R., Burgess H.A., Wolf S.G., Francis F., Chelly J., Elbaum M., Pietrokovski S. (2000). Doublecortin mutations cluster in evolutionarily conserved functional domains. Hum. Mol. Genet..

[bib42] Shahpasand K., Ahmadian S., Riazi G.H. (2008). A possible mechanism for controlling processive transport by microtubule-associated proteins. Neurosci. Res..

[bib43] Sindelar C.V., Downing K.H. (2010). An atomic-level mechanism for activation of the kinesin molecular motors. Proc. Natl. Acad. Sci. USA.

[bib44] Solecki D.J., Govek E.E., Tomoda T., Hatten M.E. (2006). Neuronal polarity in CNS development. Genes Dev..

[bib45] Song A.H., Wang D., Chen G., Li Y., Luo J., Duan S., Poo M.M. (2009). A selective filter for cytoplasmic transport at the axon initial segment. Cell.

[bib46] Tanaka T., Serneo F.F., Tseng H.C., Kulkarni A.B., Tsai L.H., Gleeson J.G. (2004). Cdk5 phosphorylation of doublecortin ser297 regulates its effect on neuronal migration. Neuron.

[bib47] Taylor K.R., Holzer A.K., Bazan J.F., Walsh C.A., Gleeson J.G. (2000). Patient mutations in doublecortin define a repeated tubulin-binding domain. J. Biol. Chem..

[bib48] Tint I., Jean D., Baas P.W., Black M.M. (2009). Doublecortin associates with microtubules preferentially in regions of the axon displaying actin-rich protrusive structures. J. Neurosci..

[bib49] Topf M., Lasker K., Webb B., Wolfson H., Chiu W., Sali A. (2008). Protein structure fitting and refinement guided by cryo-EM density. Structure.

[bib50] Tsai J.W., Lian W.N., Kemal S., Kriegstein A.R., Vallee R.B. (2010). Kinesin 3 and cytoplasmic dynein mediate interkinetic nuclear migration in neural stem cells. Nat. Neurosci..

[bib51] Verhey K.J., Hammond J.W. (2009). Traffic control: regulation of kinesin motors. Nat. Rev. Mol. Cell Biol..

[bib52] Wozniak M.J., Melzer M., Dorner C., Haring H.U., Lammers R. (2005). The novel protein KBP regulates mitochondria localization by interaction with a kinesin-like protein. BMC Cell Biol..

[bib53] Xue X., Jaulin F., Espenel C., Kreitzer G. (2010). PH-domain-dependent selective transport of p75 by kinesin-3 family motors in non-polarized MDCK cells. J. Cell Sci..

[bib54] Yonekawa Y., Harada A., Okada Y., Funakoshi T., Kanai Y., Takei Y., Terada S., Noda T., Hirokawa N. (1998). Defect in synaptic vesicle precursor transport and neuronal cell death in KIF1A motor protein-deficient mice. J. Cell Biol..

