# Supplementary material for: Molecular Basis for Specific Regulation of Neuronal Kinesin-3 Motors by Doublecortin Family Proteins
Source: Mol Cell. 2012 Sep 14;47(5):707–21. doi: 10.1016/j.molcel.2012.06.025 (PMC3549492; doi:10.1016/j.molcel.2012.06.025)
Supplement: Document S1. Supplemental Experimental Procedures, Figure S1, Figure S2, Figure S3, Figure S4, Figure S5, and Supplemental References [file mmc1.pdf]

Molecular Cell, Volume *47*

Supplemental Information

Molecular Basis for Specific Regulation of Neuronal Kinesin-3 Motors by Doublecortin Family Proteins

Judy S. Liu, Christian R. Schubert, Xiaoqin Fu, Franck J. Fourniol, Jyoti K. Jaiswal, Anne Houdusse, Collin M. Stultz, Carolyn A. Moores, and Christopher A. Walsh

Supplemental Information Inventory:

1) Supplemental Experimental Procedures and Figure Legends

Figures:
2) Supplemental Figure S1, related to Figure 1
3) Supplemental Figure S2, related to Figure 2
4) Supplemental Figure S3, related to Figure 5
5) Supplemental Figure S4, related to Figure 6
6) Supplemental Figure S5, related to Figure 7

Movies:
7) Supplemental Movie S1, related to Figure 2
8) Supplemental Movie S2, related to Figure 3
9) Supplemental Movie S3, related to Figure 4A-B
10) Supplemental Movie S4, related to Figure 4F-G
11) Supplemental Movie S5, related to Figure 5
12) Supplemental Movie S6, related to Figure 7

SUPPLEMENTAL EXPERIMENTAL PROCEDURES

Antibodies and immunostaining

The Dcx polyclonal antibody [1] and the Dclk1 polyclonal antibody [2] were previously described. The MAP2 monoclonal and MAP2 polyclonal antibodies were purchased from Sigma and Covance, respectively. Vamp2 antibody was obtained from Synaptic Systems. Kif1a antibodies were purchased from BD Transduction Laboratories (mouse monoclonal anti-Kif1a against amino acids 902-1015; BD Transduction 612094) and from Santa Cruz (goat polyclonal anti-Kif1a against amino acids 680-730; sc-19106). Cytochrome c was obtained from BD Pharmingen, and antisera to kinesin heavy chain (ab9097), GFP (ab13970), and alpha tubulin (ab6160) were purchased from Abcam. Extraction of the cytoskeleton was performed using pre-warmed BRB80 buffer (80 mM PIPES pH 6.8, 1 mM $MgCl_2$, 1 mM EGTA) with 0.1% Triton-X100 for 2 minutes at 37ºC, and cells were subsequently fixed using 4% paraformaldehyde in BRB80 buffer at 37ºC for 10 minutes, or with ice cold methanol fixation for 3 minutes followed by standard immunocytochemistry.

RNA interference constructs

The Dcx RNAi target sequence (hp) [3] was cloned into the pSilencer 1.0-U6 plasmid. Target regions for Kif1a RNAi were identified using the Broad Institute RNAi Consortium (http://www.broad.mit.edu/genome_bio/trc/publicSearchForHairpinsForm.php). Target regions for constructs 2 is ACGCAGTCTTCAACATCATT. shRNA oligos were designed using http://www.ambion.com/techlib/misc/psilencer_converter.html. The

complementary RNAi oligos were annealed and ligated into pSilencer-GFP (gift from Shirin Bonni).

Mammalian expression constructs

To tag Vamp2 with monomeric GFP (mGFP), mGFP was generated by site-directed mutagenesis of pEGFP-C1 to create the A206K mutation in EGFP, followed by in-frame cloning of the mouse Vamp2 gene to generate Vamp2-mEGFP. Subcloning Dcx into pCMV-HA from Clonetch created full-length HA-Dcx. HA-tagged Dcx linker mutants W146C and K174E were created using Stratagene's QuikChange Site-Directed Mutagenesis kit. For the coarse mapping of the interaction, HEK cells were transfected with full length Kif1a, or Kif1a (1-396) [4] or the motor domain of Kif1a (amino acids 1-365) which was sub-cloned in frame with GFP in the pCAG vector.

Cloning of human Dcx and Kif1a and other constructs for bacterial expression

Human Dcx (amino acids 1-360) and Kif1a (amino acids 1-361), including a C-terminal His_{8x}-tag followed by a triple stop codon, were cloned into pFN18A HaloTag T7 Flexi expression vectors (Promega) using *AsiSI* and *PmeI* restriction sites. Dcx linker mutants W146C and K174E were created using QuikChange Site-Directed Mutagenesis kit (Stratagene). In addition, human Dcx (amino acids 1-360) was cloned into the pET-45b(+) expression vector (Novagen) for Halo-tag free expression. Restriction digestion and Sanger sequencing verified all constructs. Kif1a (C351)-containing pET vector [5] was obtained from Dr. Nobutaka Hirokawa (University of Tokyo, Japan).

Protein expression and purification

All bacterial expression constructs were transfected into *E. coli* strain BL21(DE3)pLys (Novagen), grown to $OD_{600nm} \sim 0.5$ at 37°C in 2xYT medium, induced

by addition of 0.25 mM isopropyl β-d-thiogalactoside (IPTG), and grown for an additional 4 hours to overnight at 24°C. Cells from a 1 L culture were harvested by centrifugation and frozen at -20°C; thawed pellets were resuspended in 25 ml cold Ni-NTA binding buffer (10 mM Tris-HCl pH 8.5, 500 mM NaCl, 60 mM imidazole, 1 mM DTT) supplemented with 1X Complete Mini (EDTA-free) Protease Inhibitor Cocktail Tablets (Roche), 1.25 mg lysozyme and 62.5 U DNase I (Pierce). After incubation for 30-60 min at room temperature (shaking or rocking), the cells were sonicated at powers of 20-25 W on ice in 10 s bursts for a total of 2 minutes. After clarification by centrifugation > 15,000 x g, the supernatant was collected and filtered through a 0.45-micron pore size syringe filter. The filtered supernatant was loaded onto a 1 ml His GraviTrap affinity purification column, pre-equilibrated with 10 column volumes (CVs) of cold Ni-NTA binding buffer, and connected to a LabMate PD-10 buffer reservoir (GE LifeSciences). After washing with 15 CVs cold Ni-NTA binding buffer, the proteins were eluted with 4-8 ml of Ni-NTA elution buffer (10 mM Tris-HCl pH 8.5, 500 mM NaCl, 500 mM imidazole, 1 mM DTT) and immediately exchanged into appropriate storage buffers (Dcx storage buffer: 50 mM Tris-HCl pH 8.0, 150 mM NaCl, 1 mM DTT; Kif1a storage buffer: 10 mM MOPS pH 7.0, 100 mM NaCl, 1 mM EGTA, 1 mM $MgCl_2$, 20 % w/v sucrose) using Zeba Spin Desalting Columns (Pierce, 7K MWCO, 10 ml column volume). Samples were concentrated using Amicon Ultra-4 Centrifugal Filter Units (Millipore, 10 kDa MWCO). Sample purity was assessed by SDS-PAGE followed by staining with Imperial Protein Stain (Pierce). After concentration, samples were aliquoted and stored at -80°C.

In utero electroporation

Briefly, pregnant Swiss Webster or *Dclk-/-* mice were euthanized at E15 and embryos removed at E14.5 or E15.5. The lateral ventricle was microinjected with 1 microliter of shRNAi constructs at concentrations of 1.5-2 micrograms per microliter (scrambled control, Kif1a or Dcx) using pulled glass microcapillary needles with plasmids in a 0.01% fast green solution (Sigma). Electrodes were placed on either side of the embryo's head and 5x 100 ms square pulses at 40 V were administered at 950 ms intervals using a BTX830 square-wave pulse generator (Genetronics, Havard Apparatus), vibratome-sectioned for cortical slice culture [6] on Millicell inserts for 6-well plates for 72 hours and then fixed for 5 hours in 4% paraformaldehyde prior to immunostaining and imaging.

Multiple sequence alignment of tandem DCX domain proteins

Amino acid sequences encoding the linker between the R1 and R2 domain of selected tandem DCX proteins were chosen as defined in UniProt (http://www.uniprot.org). The alignment was performed using Kalign 2.0 [7] (freely available at http://www.ebi.ac.uk/Tools/kalign) with the following input parameters: gap open penalty = 2.0, gap extension penalty = 0.85, terminal gap penalties = 0.45, bonus score = 0.

MT binding and co-immunoprecipitation from cortical and HEK cell lysates

MT binding was assayed by transfecting HEK 293T cells with the kinesin constructs stated above with or without Dcx. For MT binding assays, lysates made in BRB80/0.1% Triton X-100 buffer were brought to 37°C and taxol was added to 20 μM, AMP-PNP (5,9-adenylylimidodiphosphate) or ATP to 2.5 mM, and taxol-stabilized MTs

to 0.1 mg/ml (except for the endogenous pull-down where no exogenous MTs were added). After 30-45 min incubation, lysates were overlaid on 1/2 volume of 10% sucrose in BRB80 buffer with 20 μM taxol. MTs were sedimented in a Sorvall tabletop ultracentrifuge for 30 min at > 100,000 x g at 37°C. Quantitation was performed by normalizing Kif1a to the Kif1a expression in the lysate followed by calculating the bound fraction in the pellet as P/(S+P). Statistical analysis of these experiments was performed using the Mann-Whitney or Student's t-test. Endogenous MT binding assays from brain lysates were performed in the same manner except that no exogenous tubulin was added and taxol and GTP were added to a final concentration of 2 mM and 1mM, respectively, to 100 μl of brain lysate. All lysates were overlaid on a 30% sucrose cushion prior to ultracentrifugation.

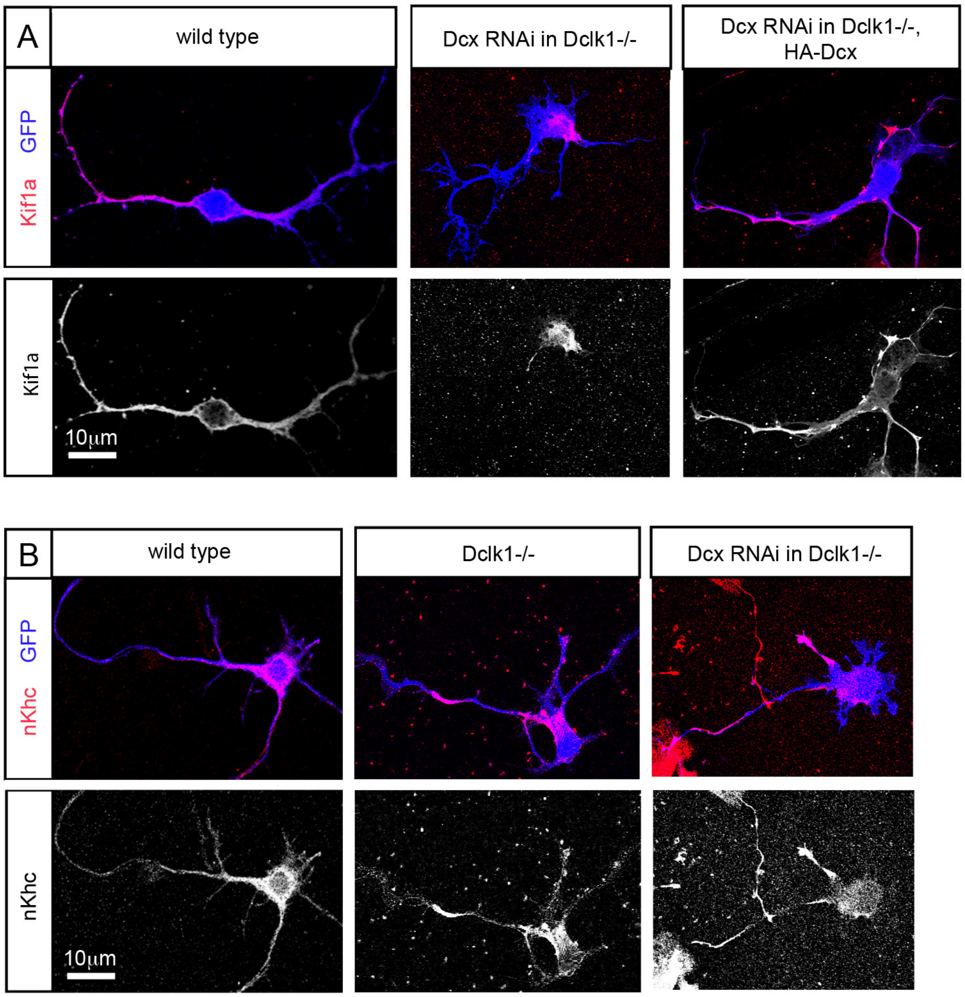

Figure S1 (related to main figure 1): Kif1a and conventional kinesin localization in Dcx/Dclk1 deficient neurons. (A) Antisera against amino acids 680-730 of Kif1a shows the same pattern of expression as the antiserum against amino acids 902-1015 (used in Figure 1), with Kif1a retention in the cell body in the double deficient Dcx/Dclk1 neurons and markedly less Kif1a in the neurites. (B) Immunostaining for neuronal kinesin heavy chain (nKhc) demonstrates signal in the cell body and neurites in both WT and mutant neurons. Scale bars are 10μm.

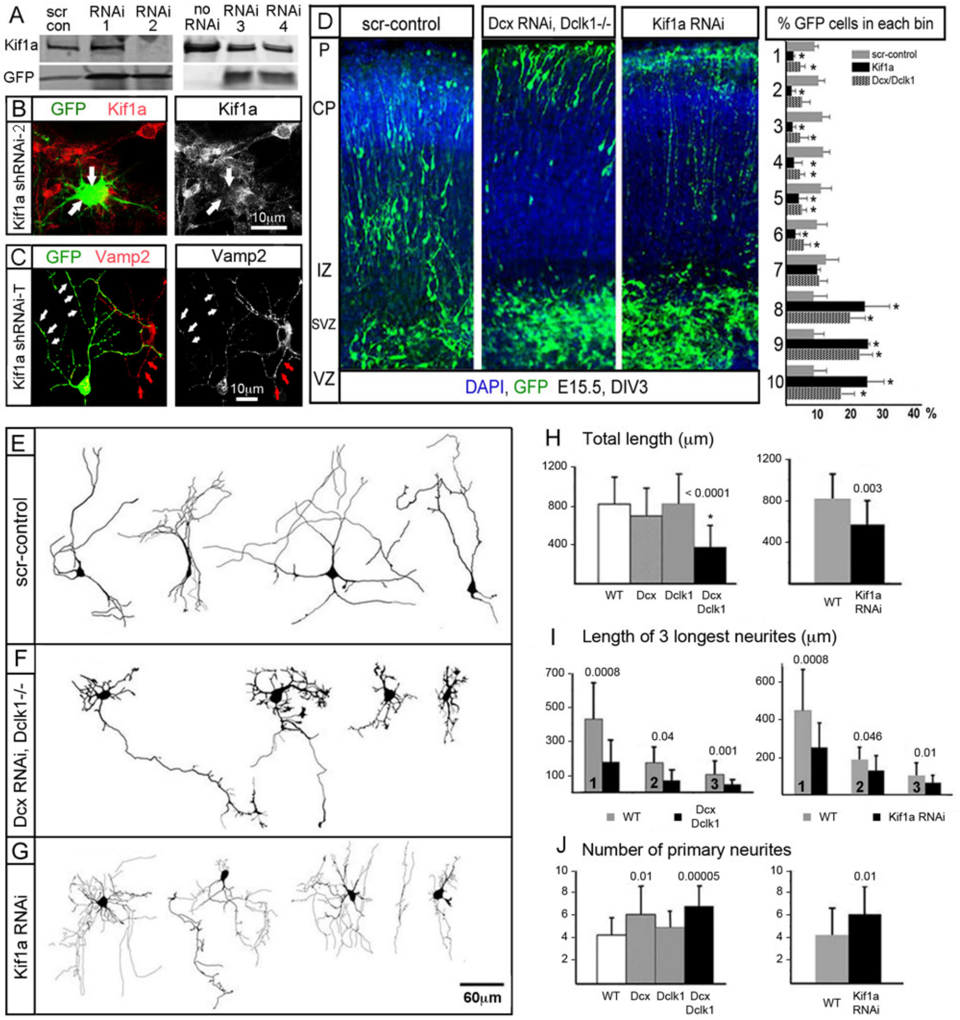

Figure S2 (related main figure 2): Knockdown of Kif1a phenocopies Dcx/Dclk1 deficiency in neurons. (A) Four shRNAi constructs (1-4) to Kif1a were tested along with a scrambled control (scr con) and a condition without co-transfection of shRNAi (no RNAi) in HEK cells. Transfection of exogenous Kif1a with four different RNAi constructs in vectors co-expressing GFP as a marker for transfection demonstrates nearly complete knockdown of Kif1a with construct 2 and about 50% with 3 and 4. No reduction in expression is observed by construct 1. (B) Expression of the Kif1a shRNAi #2 construct (green) knocks down endogenous Kif1a expression in neurons (red). (C) An Kif1a shRNAi (T) designed to interact with a previously published sequence [8] has the

same phenotype as that in Figure 2B and results in decreased Vamp2 in neurites and accumulation in the soma. (C) Radial migration is assayed in slice cultures from E15.5 embryos. WT or *Dclk1-/-* embryos were microinjected in the lateral ventricle with directed electroporation toward the cortical ventricular zone either using an empty vector control, Kif1a RNAi, or Dcx RNAi in *Dclk1-/-* mutant embryos. Examined 96 hours later, the graph shows the percent of cell bodies in evenly divided regions of the cortex (1-10) from the pia to the lateral ventricle, illustrating migration in Dcx/Dclk1 and Kif1a deficient neurons. Asterisks denote statistically significant p-values < 0.05. (D-F) WT or *Dclk1-/-* dissociated neurons from E14 embryos were transfected with empty vector, Kif1a, or Dcx RNAi construct, respectively. Tracings of WT (D), Dcx/Dclk1 deficient neurons (E) and Kif1a knockdown neurons (F) at DIV5 are shown. WT, Dcx, *Dclk1-/-*, Dcx/Dclk1 deficient and Kif1a RNAi-treated neurons are compared in terms of total neurite length (G), the lengths of the first, second, and third longest neurite of WT (H), and number of primary neurites arising from the cell body (I). In all panels error bars represent the SEM. P-values of significant comparisons are shown.

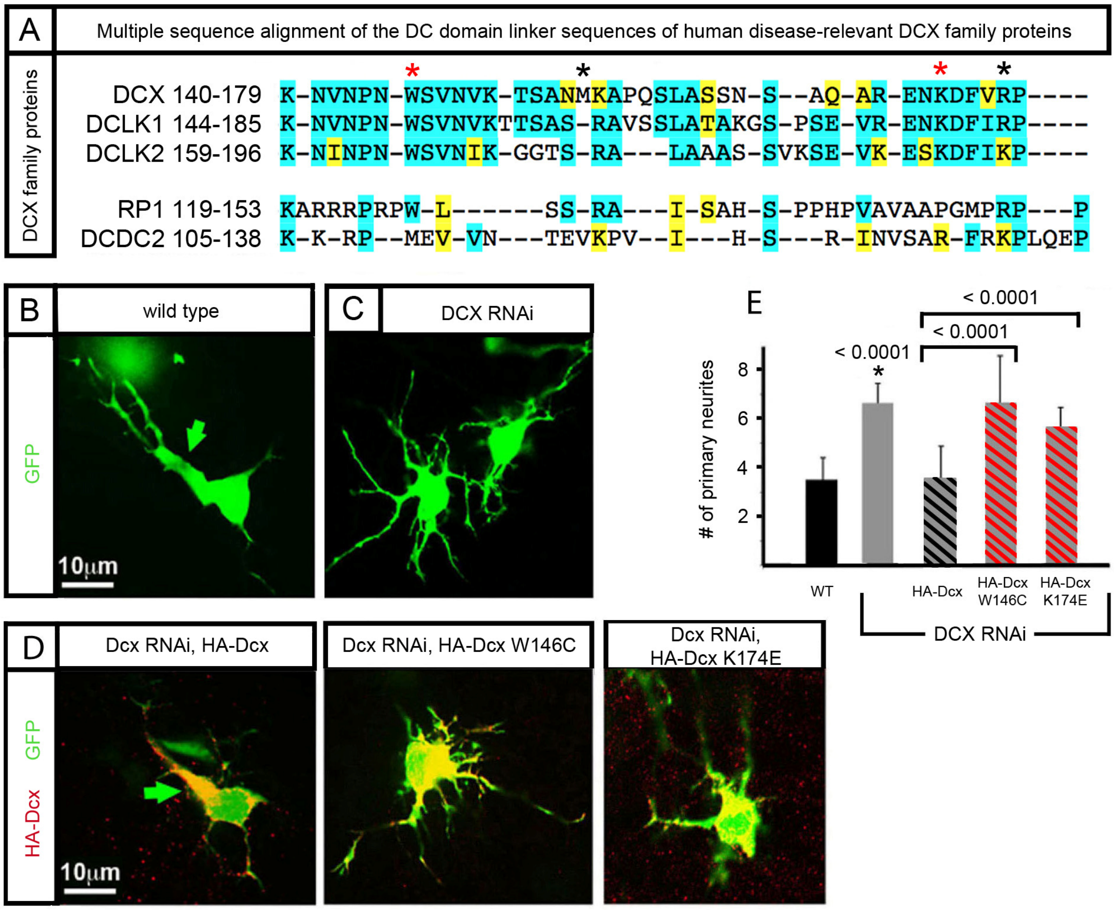

Figure S3 (related to main figure 5): Point mutations in the Dcx linker fail to rescue the Dcx RNAi phenotype. (A) Alignment of linker sequences in tandem Dcx family proteins (Dcx, Dclk1, Dclk2, RP1, and Dcdc2) shows conservation of residue identity in at least two closely related sequences (cyan shading). Yellow shading indicates conservation of residue property, such as charge, hydrophobicity, or polarity. Asterisks indicate the following known patient mutations in the human Dcx protein: W146C, K174E, R178C, R178L with red asterisks indicating W146C and K174E. (B) In dissociated cortical cultures, a control neuron transfected with GFP is shown with an apical dendrite (green arrow) and typical pyramidal morphology. (C) Knockdown of Dcx by RNAi results in an increase in the number of primary neurites and a lack of apical

dendrite formation. (D) A neuron transfected with Dcx RNAi is rescued by expression of HA-tagged WT Dcx (red) and has an apical dendrite (green arrows), but the linker mutants Dcx W146C and Dcx K174E fail to rescue the Dcx RNAi neurite phenotype. (E) Quantification of the number of primary neurites for RNAi and rescue with either WT or mutant Dcx W146C or K174E shows significant differences from WT neurons. Error bars represent SEM and p-values shown (two tailed t-test). Scale bars represent 10 μm for all panels (B-D).

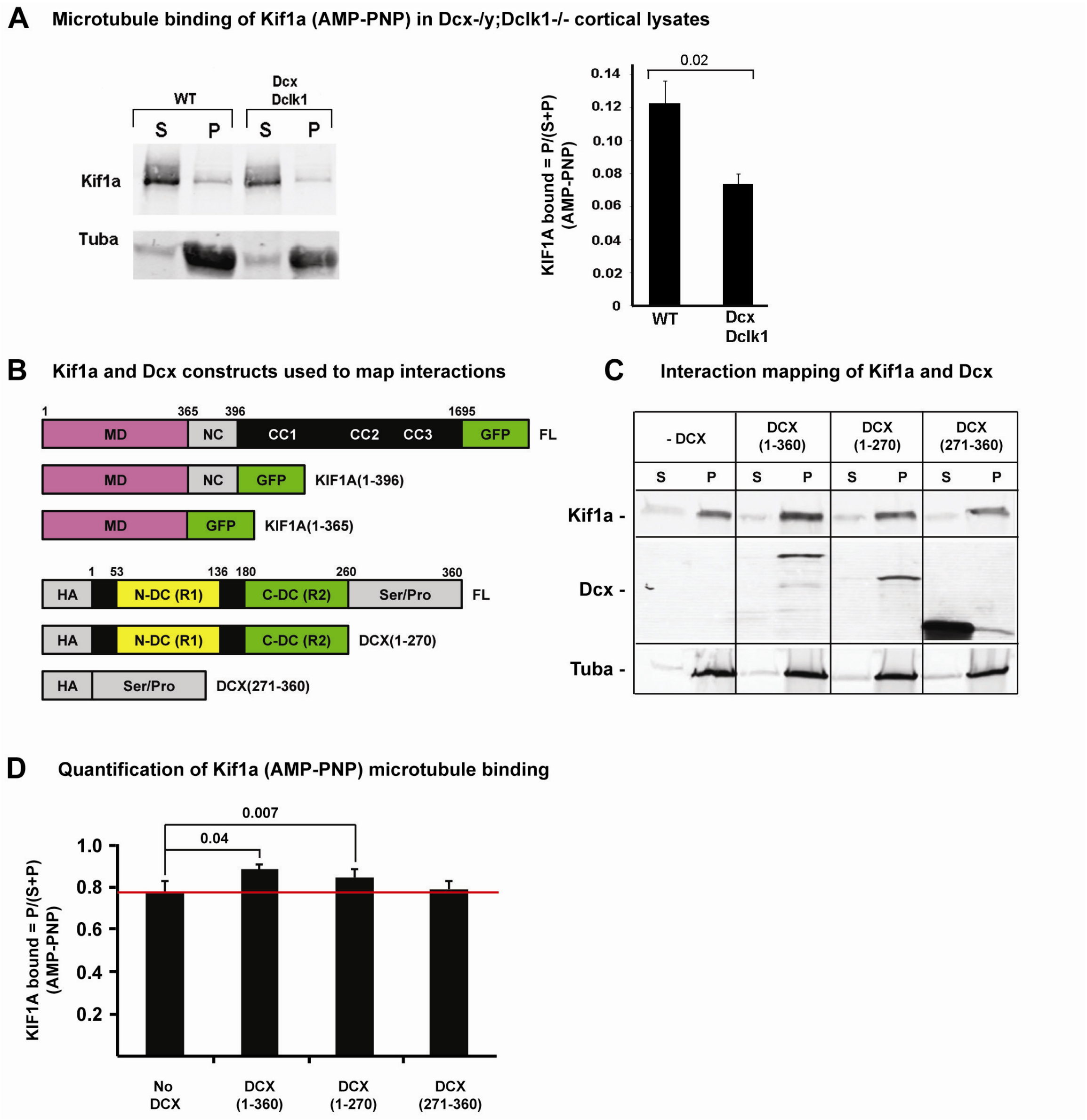

Figure S4 (related to main figure 6): Microtubule binding assays with AMP-PNP shows increased association of Kif1a to microtubules in presence of Dcx. (A) MT binding assay on cortical lysates prepared from WT and Dcx-/y; Dclk1-/- at postnatal day 0 (P0) shows binding of endogenous Kif1a to polymerized MTs in the pellet (P) and the unbound fraction in the supernatant (S). Graph shows the increase of Kif1a in pellet over baseline calculated as % bound = P/(S+P). Error bars represent SEM and p-values are shown (two-tailed t-test, n = 3). (B) Truncation constructs of Kif1a and Dcx that were

used to map (C) and quantify (D) the Dcx-Kif1a interaction domains using MT co-sedimentation assays after overexpression in HEK cells. (C) Binding of the Kif1a motor domain (MD) to MTs is shown in presence of the N-terminal (amino acids 1-270, including N-DC (R1) and C-DC (R2)) or C-terminal (amino acids 271-361, including Ser/Pro-rich) domains of Dcx. (D) Graph shows the increase of Kif1a in pellet over baseline calculated as % bound = P/(S+P). Error bars represent SEM and p-values are shown (two-tailed t-test, n = 5).

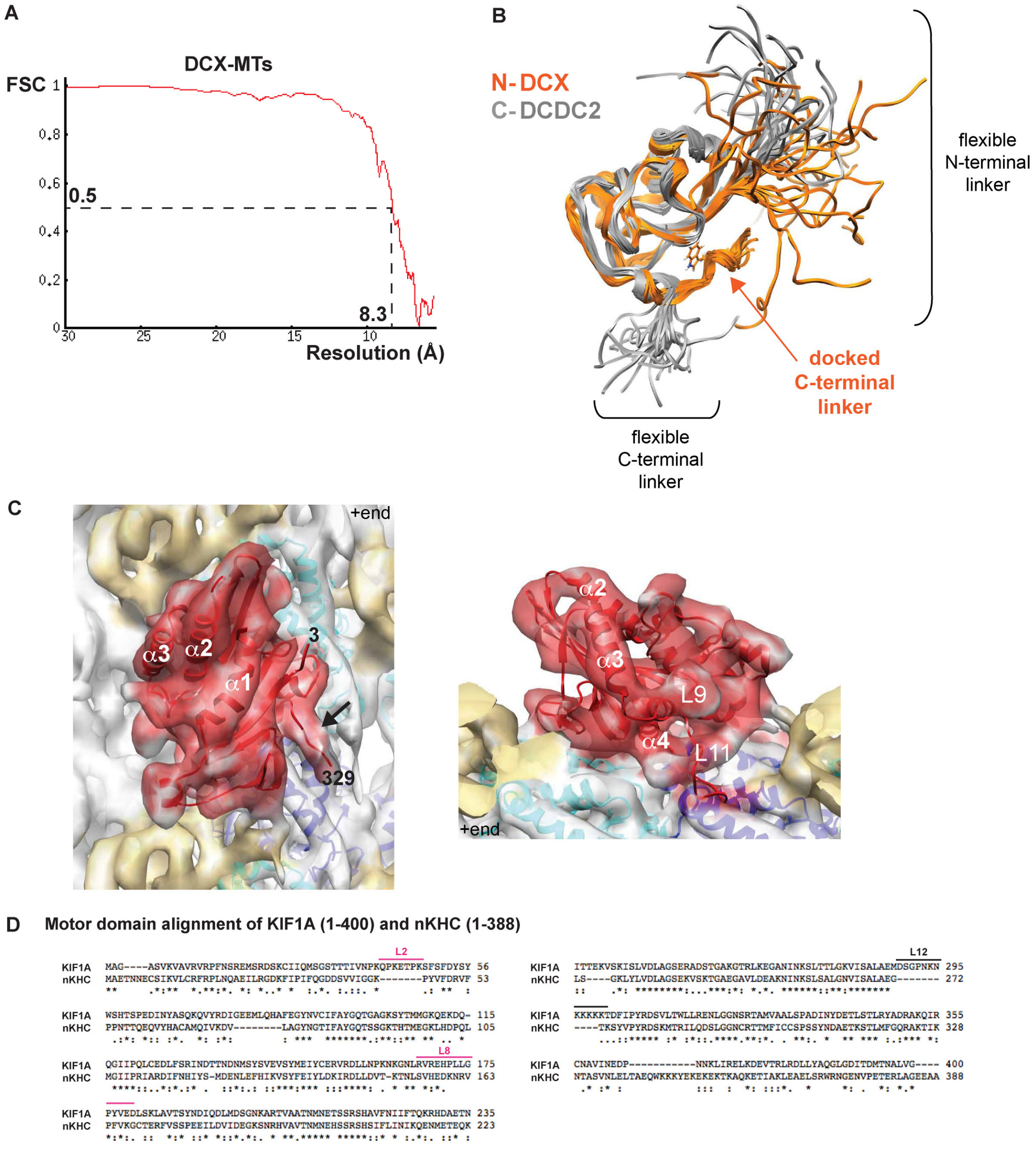

Figure S5 (related to main figure 7): Docking of Dcx and Kif1a bound to the tubulin dimer. (A) Fourier Shell Correlation (FSC) curve calculated for two independent cryo-EM reconstructions of Dcx-MTs in absence of kinesin. A resolution of 8.3 Å was determined following the FSC 0.5 criterion. (B) The superimposition of NMR models of Dcx R1 (1MJD.PDB; [9]) and Dcdc2 R2 (2DNF.PDB) illustrates how the linker C-

terminal of R1 docks onto the globular domain via W146, whereas it is not docked in R2. Dcdc2 R2 has 32% sequence identity with Dcx R2 (27% with Dcx R1) and like Dcx R2 it does not have a residue equivalent to W146 in its C-terminal linker. The conformation of the C-terminal linker seen in our cryo-EM map of Dcx-MTs without kinesin (Figure 7B) is consistent with assignment of the DC domain density bound to the MT as being R1. (C) 8.2 Å resolution cryo-electron microscopy reconstruction of Dcx-MTs co-decorated with conventional kinesin motor domain (T93N) with no nucleotide bound (transparent surface, tubulin colored in grey, kinesin in red, DCX in yellow; [10]) docked with atomic coordinates (ribbons) of tubulin (2XRP.PDB, alpha in blue, beta in cyan), DCX R1 (1MJD.PDB, orange) and conventional kinesin motor domain (1BG2.PDB, amino acids 3-323, red; cross-correlation value of 0.746 for the fit). The neck-linker of the nucleotide-free motor is clearly resolved in the cryo-EM map (arrow, left panel), pointing towards the minus end of the MT, in an alternative docked conformation not seen previously [11]. The neck-linker (amino acids 324-329), loops L1, L9 and helix α6 were modeled into the EM density as described in Experimental Procedures. This improved the fit, with a cross-correlation score of 0.769. (D) Pairwise sequence alignment of the motor domains of human Kif1a (GI: 119364606) and nKhc (GI: 143811412). The alignment was performed using ClustalW2 (http://www.ebi.ac.uk/Tools/msa/clustalw2/) with default settings. Loops L2 and L8 are highlighted in pink. In addition, the K-loop (L12) is highlighted in black. Most significantly, L2 shows an insertion of 7 residues unique to Kif1a, which marks this loop as a potential interaction site with Dcx that might confer specificity to this unique MAP-motor pair.

SUPPLEMENTAL REFERENCES

1. Gleeson JG, Lin PT, Flanagan LA, Walsh CA: **Doublecortin is a microtubule-associated protein and is expressed widely by migrating neurons**. *Neuron* 1999, **23**(2):257-271.
2. Deuel TA, Liu JS, Corbo JC, Yoo SY, Rorke-Adams LB, Walsh CA: **Genetic interactions between doublecortin and doublecortin-like kinase in neuronal migration and axon outgrowth**. *Neuron* 2006, **49**(1):41-53.
3. Bai J, Ramos RL, Ackman JB, Thomas AM, Lee RV, LoTurco JJ: **RNAi reveals doublecortin is required for radial migration in rat neocortex**. *Nat Neurosci* 2003, **6**(12):1277-1283.
4. Hammond JW, Cai D, Blasius TL, Li Z, Jiang Y, Jih GT, Meyhofer E, Verhey KJ: **Mammalian Kinesin-3 motors are dimeric in vivo and move by processive motility upon release of autoinhibition**. *PLoS Biol* 2009, **7**(3):e72.
5. Kikkawa M, Sablin EP, Okada Y, Yajima H, Fletterick RJ, Hirokawa N: **Switch-based mechanism of kinesin motors**. *Nature* 2001, **411**(6836):439-445.
6. Polleux F, Ghosh A: **The slice overlay assay: a versatile tool to study the influence of extracellular signals on neuronal development**. *Sci STKE* 2002, **2002**(136):pl9.
7. Lassmann T, Sonnhammer EL: **Kalign--an accurate and fast multiple sequence alignment algorithm**. *BMC Bioinformatics* 2005, **6**:298.
8. Tsai JW, Lian WN, Kemal S, Kriegstein AR, Vallee RB: **Kinesin 3 and cytoplasmic dynein mediate interkinetic nuclear migration in neural stem cells**. *Nat Neurosci* 2010, **13**(12):1463-1471.
9. Kim MH, Cierpicki T, Derewenda U, Krowarsch D, Feng Y, Devedjiev Y, Dauter Z, Walsh CA, Otlewski J, Bushweller JH *et al*: **The DCX-domain tandems of doublecortin and doublecortin-like kinase**. *Nat Struct Biol* 2003, **10**(5):324-333.
10. Fourniol FJ, Sindelar CV, Amigues B, Clare DK, Thomas G, Perderiset M, Francis F, Houdusse A, Moores CA: **Template-free 13-protofilament microtubule-MAP assembly visualized at 8 A resolution**. *J Cell Biol* 2010, **191**(3):463-470.
11. Sindelar CV, Downing KH: **The beginning of kinesin's force-generating cycle visualized at 9-A resolution**. *J Cell Biol* 2007, **177**(3):377-385.
