# Supplementary figures and images for: Molecular Basis for Specific Regulation of Neuronal Kinesin-3 Motors by Doublecortin Family Proteins
Source: Mol Cell. 2012 Sep 14;47(5):707–21. doi: 10.1016/j.molcel.2012.06.025 (PMC3549492; doi:10.1016/j.molcel.2012.06.025)

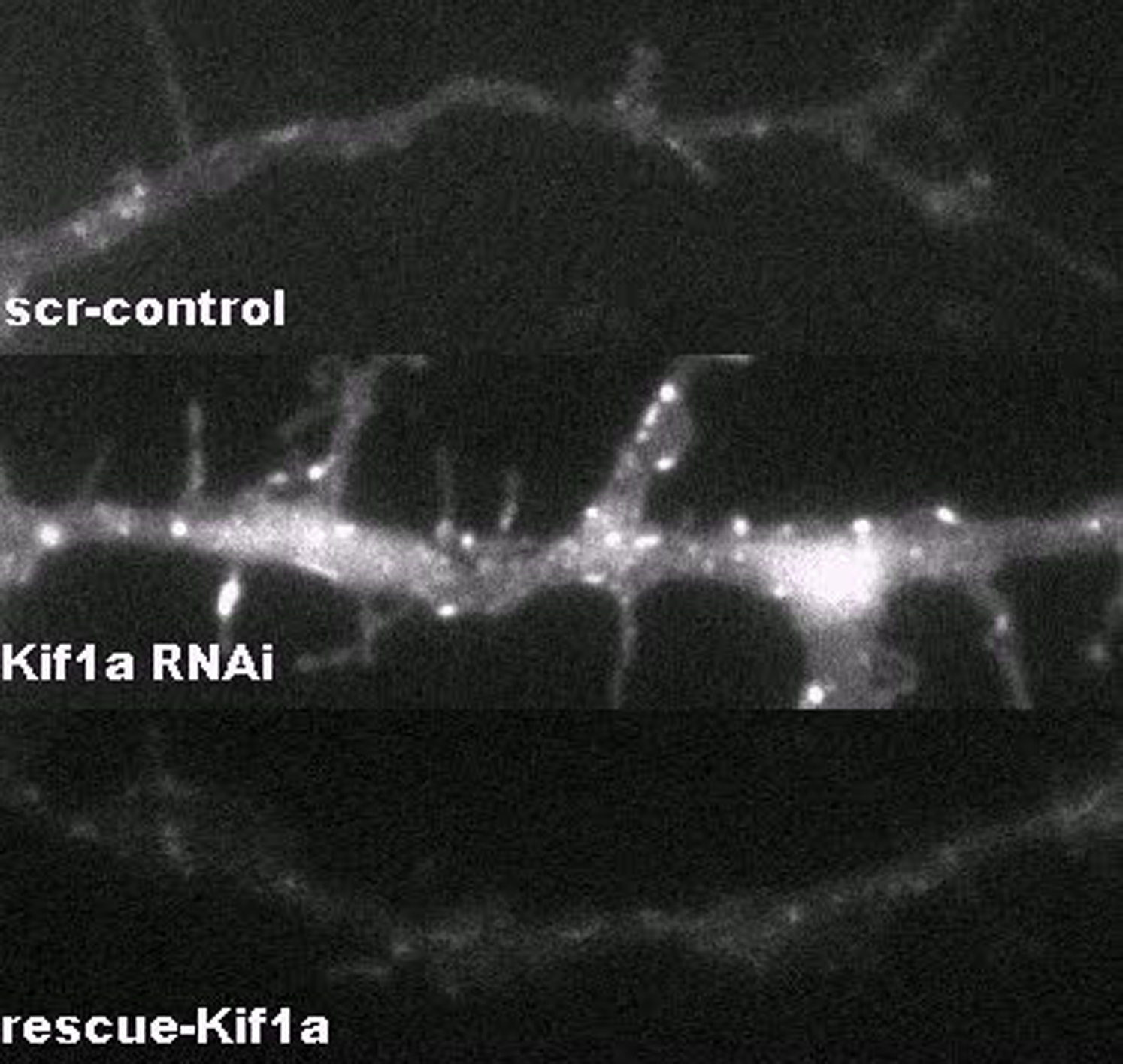

Supplement: Movie S1. Transport of Vamp2-GFP in Kif1a RNAi-Treated Neurons — On dissociation, neurons are treated with a scrambled control, Kif1a shRNAi, or Kif1a shRNAi and myc-tagged human Kif1a (rescue). Neurons are transfected with Vamp2-GFP at 3 DIV and imaged at 4 DIV. Frames are acquired at one frame per second for 120 s. (Related to Figure 2.) [file mmc2.jpg]

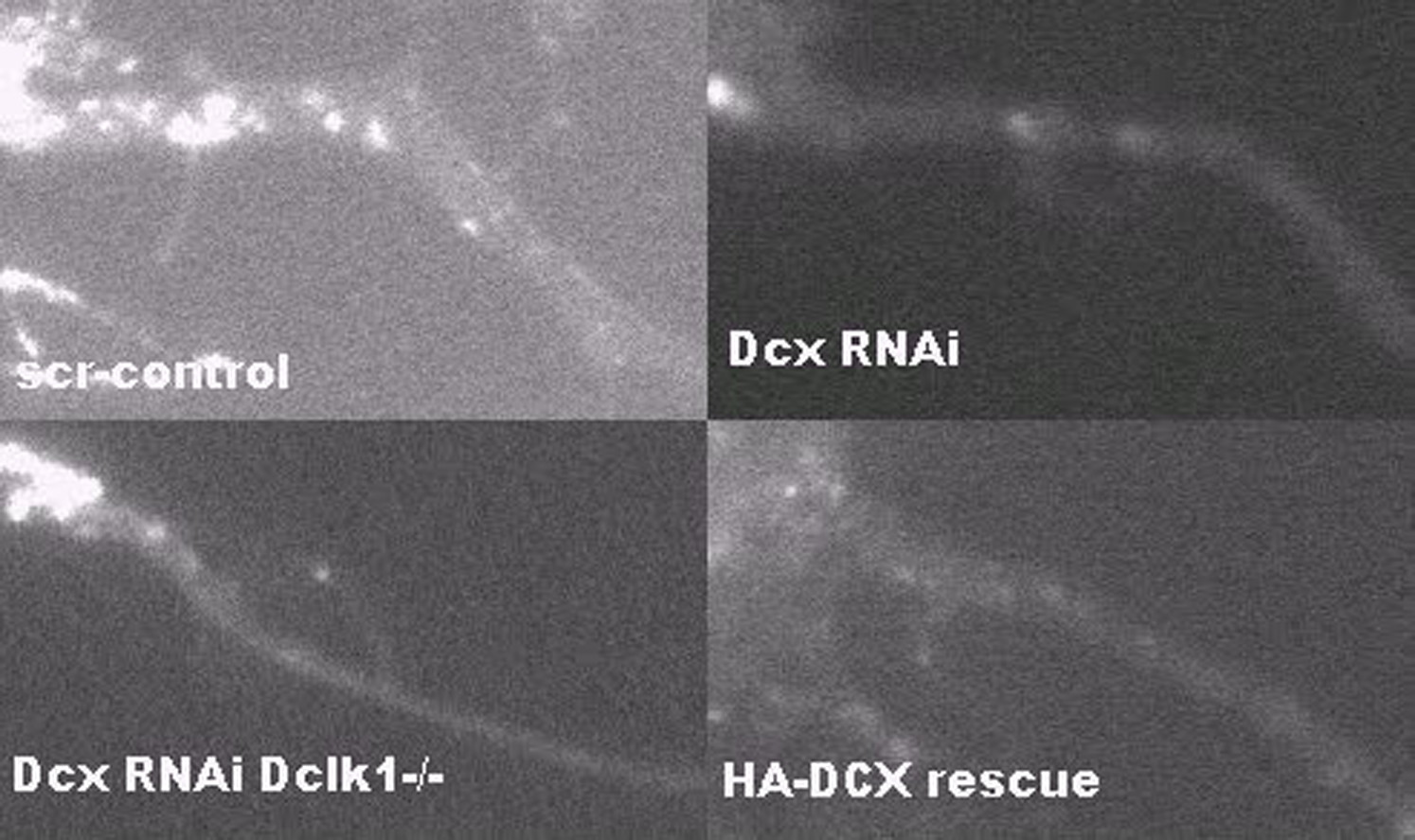

Supplement: Movie S2. Efflux of Vamp2-GFP from the Cell Body into Neurites of Dcx/Dclk1-Deficient Neurons — At the time of dissociation, wild type or Dclk1−/− neurons are transfected with a scrambled control, Dcx shRNAi, or the Dcx shRNAi and an HA-tagged, RNAi-resistant Dcx (rescue). Neurons are transfected with Vamp2-GFP at 3 DIV and imaged at 4 DIV. Frames are acquired at one frame per second for 120 s. (Related to Figure 3.) [file mmc3.jpg]

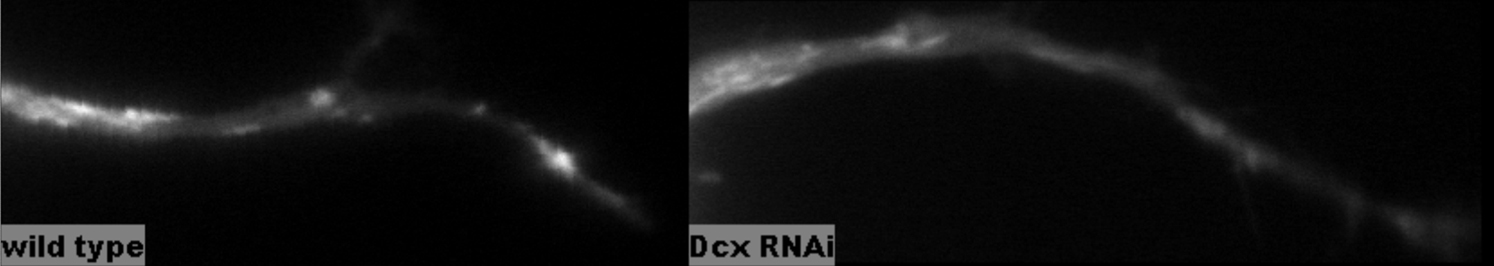

Supplement: Movie S4. Transport of Mito-RFP in Neurites of Dcx-Deficient Neurons — Wild-type neurons are transfected with a scrambled control or an shRNAi Dcx construct at the time of dissociation. Plated neurons are transfected with Mito-DsRed at 4 DIV and imaged at 5 DIV. Frames are acquired at one frame per second for 120 s. (Related to Figure 4.) [file mmc5.jpg]

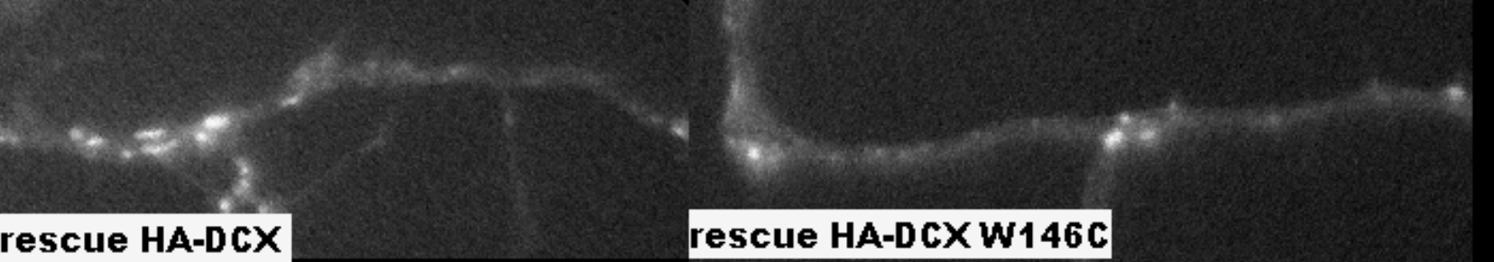

Supplement: Movie S5. Transport of Vamp2-GFP in Dcx RNAi-Treated Neurons Rescued with HA-Dcx W146C — Wild-type neurons are transfected with Dcx shRNAi and a HA-tagged Dcx rescue construct or an HA-tagged Dcx W146C mutant construct. Neurons are transfected with Vamp2-GFP at 3 DIV and imaged at 4 DIV. Frames are acquired at one frame per second for 120 s. (Related to Figure 5.) [file mmc6.jpg]

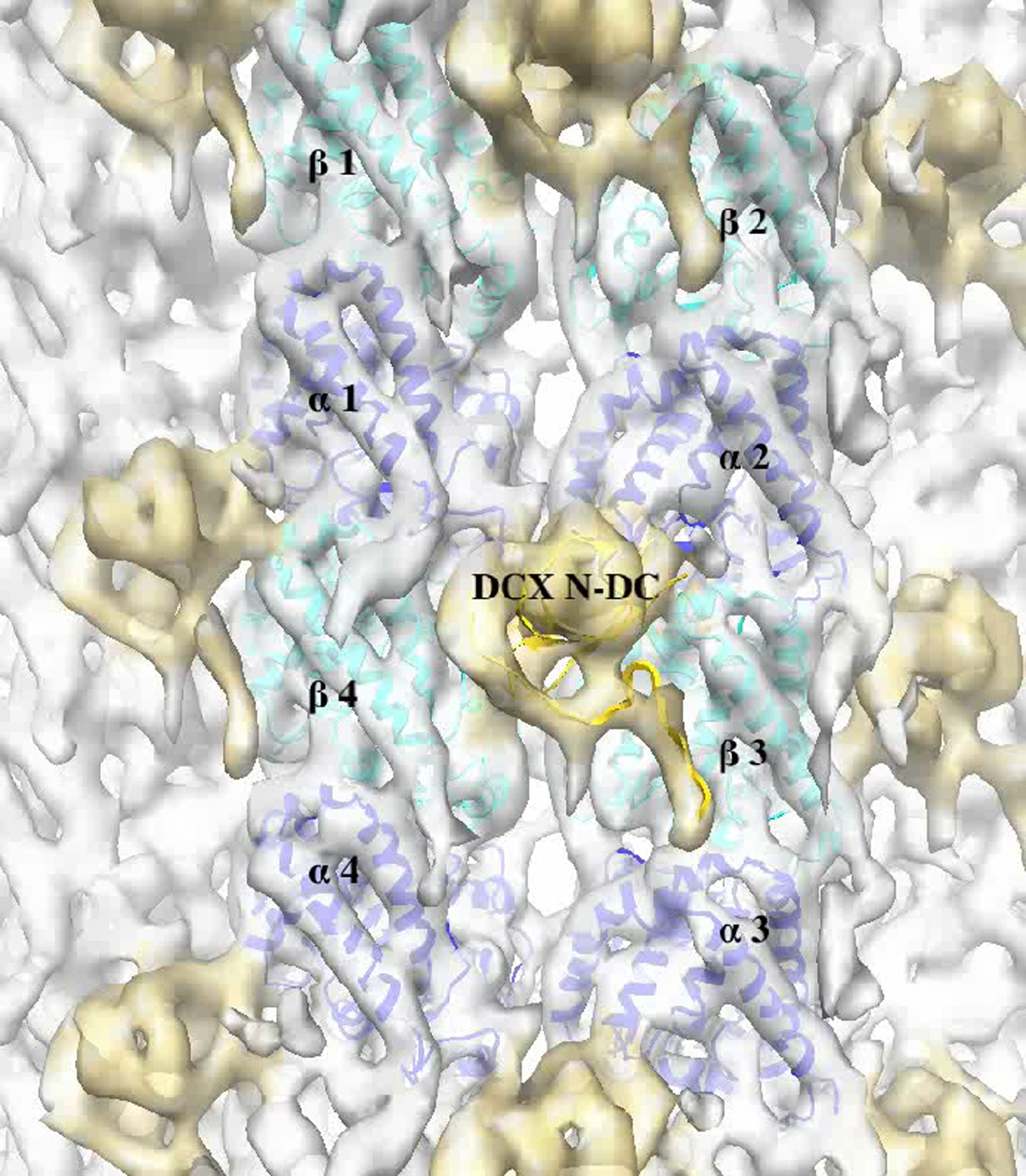

Supplement: Movie S6. Models of Dcx Binding to Microtubules in the Presence and Absence of Kinesin Highlight Conformational Rearrangements in the Bound Dcx — Cryo-EM maps, fitted atomic coordinates, and rendering parameters as in Figure 7. [file mmc7.jpg]
